# Skeletal muscle mitochondrial–methylation–neurotransmitter crosstalk: a novel synergistic model of betaine, tyrosine, and Cordyceps for exercise performance in older adults, individuals with metabolic disorders, and healthy populations

**DOI:** 10.3389/fnut.2026.1812286

**Published:** 2026-08-18

**Authors:** Chunhong Xu, Weilu Cai, Jing Li, Ying Ma

**Affiliations:** 1 Physical Education Teaching and Research Department, General Education College, Xinjiang University of Technology, Hotan, Xinjiang, China; 2 Department of Physical Education, Baoding University of Technology, Baoding, Hebei, China; 3 Department of Physical Education and Teaching Research, Xinjiang College of Science and Technology, Korla, Xinjiang, China

**Keywords:** betaine, body composition, Cordyceps, exercise, mitochondrial bioenergetics, muscle fatigue biomarkers, tyrosine

## Abstract

Athletic performance and exercise adaptation are influenced by intricately coordinated interactions among mitochondrial bioenergetics, methylation-dependent metabolic pathways, and the regulation of central neurotransmitters. The potential of Cordyceps-based supplementation to enhance mitochondrial ATP production, optimize oxygen utilization, and improve fatigue resistance has been extensively studied. Nevertheless, the exploration of its integration with complementary metabolic modulators remains inadequately addressed. This review introduces an innovative systems-level model that synthesizes Cordyceps with betaine and tyrosine, aiming to target both peripheral and central determinants of athletic performance. Betaine acts as a methyl donor and osmolyte, facilitating creatine synthesis, enhancing cellular hydration, and promoting metabolic resilience, thereby improving muscle power output and fostering adaptations in body composition. Tyrosine, serving as a precursor for catecholamines, significantly influences dopaminergic and noradrenergic signaling, with the potential to mitigate central fatigue and enhance cognitive endurance in the presence of physiological stress. We propose a comprehensive integrative framework of mitochondrial–methylation–neurotransmitter interactions to elucidate how these agents may collectively augment ATP efficiency, redox equilibrium, neuromuscular performance, and recovery processes. Notably, this model may possess translational implications not solely for healthy athletes but also for elderly populations and individuals experiencing metabolic dysfunction, wherein compromised mitochondrial activity and neurotransmitter signaling play a pivotal role in diminished exercise capacity. Subsequent mechanistic and clinical investigations are essential to substantiate this multi-faceted supplementation approach aimed at precision performance enhancement.

## 1 Introduction

Athletic performance represents a multifaceted phenotype influenced by the interplay of mitochondrial bioenergetics, epigenetic modulation, and neurotransmitter signaling (1). These biological systems do not function in isolation; instead, they constitute a dynamic and reciprocal network that influences cellular energy availability, metabolic adaptability, neuromuscular efficiency, and cognitive-motivational dynamics. In aging populations, among individuals afflicted with metabolic disorders, and even within healthy individuals subjected to elevated physiological demands, the dysregulation of this intricate network contributes to diminished endurance, impaired recovery processes, sarcopenia, insulin resistance, and cognitive decline (2, 3). Emerging empirical evidence indicates that a simultaneous approach targeting mitochondrial function, one-carbon metabolism, and catecholaminergic pathways may yield a more efficacious strategy than isolated interventions (4). Mitochondrial dysfunction represents a critical characteristic of both the aging process and metabolic disorders, including type 2 diabetes and obesity (5). Extensive research has documented age-related reductions in mitochondrial biogenesis, diminished oxidative phosphorylation efficiency, and an increase in the production of reactive oxygen species (ROS), particularly within skeletal muscle and neural tissues (6). In a study, it was reported a significant correlation between age-induced mitochondrial deterioration and modifications in NAD^+^ metabolism along with epigenetic regulatory mechanisms (7). Additionally, compromised mitochondrial dynamics and diminished PGC-1α signaling have been shown to lead to a reduction in endurance capacity and metabolic flexibility (8). In the context of metabolic disorders, mitochondrial inefficiency serves to amplify lipid accumulation, inflammation, and insulin resistance (9). However, it must be explicitly noted that the vast majority of performance-focused supplementation data generated to date relies heavily on young, healthy, trained cohorts. Extrapolating these findings to clinical or geriatric populations represents a substantial translational leap that remains largely unvalidated by direct clinical trials.

Epigenetic regulation, with a particular emphasis on DNA methylation, constitutes a critical dimension that governs metabolic and neuromuscular adaptations. The patterns of DNA methylation exhibit a responsiveness to variables such as nutritional status, physical activity, and metabolic stress, thereby influencing genes that are integral to mitochondrial biogenesis, inflammation, and neurotransmission. The hypomethylation of metabolic genes induced by exercise serves to augment oxidative capacity (10). The metabolic pathway of one-carbon metabolism, which regulates the availability of methyl groups via S-adenosylmethionine (SAM), is fundamental to this regulatory mechanism. Nutritional components like betaine function as methyl donors, facilitating the remethylation of homocysteine and upholding methylation homeostasis. The dysregulation of methylation pathways has been associated with the onset of metabolic syndrome, cognitive decline, and muscular dysfunction (11). Neurotransmitter signaling, especially the dopaminergic and noradrenergic systems, plays an essential role in motivation, motor coordination, reaction time, and perceived exertion. Tyrosine, which serves as a precursor to catecholamines, has demonstrated efficacy in facilitating cognitive performance during stressful situations and enhancing neuromuscular activation in challenging environments (12). Dopamine signaling engages with mitochondrial function at various levels, as the metabolism of dopamine affects oxidative stress, while the production of adenosine triphosphate (ATP) by mitochondria is crucial for neurotransmitter synthesis and vesicular transport (13). The process of aging, along with metabolic disorders, is often correlated with compromised dopaminergic signaling, which contributes to diminished physical performance and increased levels of fatigue. Natural bioactive compounds, including species from the genus Cordyceps, especially Cordyceps sinensis and Cordyceps militaris, have attracted scholarly attention due to their potential efficacy in enhancing mitochondrial ATP synthesis, optimizing oxygen utilization, and bolstering antioxidant defenses. Cordycepin, along with analogous nucleoside derivatives, may serve to activate AMP-activated protein kinase (AMPK) and augment mitochondrial operational efficiency, concurrently modulating both inflammatory and redox signaling pathways (14, 15). Initial investigations involving human subjects indicate potential enhancements in aerobic capacity and resistance to fatigue; however, a comprehensive mechanistic integration with methylation processes and neurotransmitter signaling pathways remains inadequately explored.

Although a significant body of evidence substantiates the discrete functions of mitochondrial optimization, methylation facilitation, and catecholamine enhancement, there exists a paucity of studies that have endeavored to establish a cohesive framework unifying these systems. Betaine facilitates the equilibrium of methylation and provides osmoprotection; tyrosine augments the synthesis of catecholamines and enhances neuromuscular drive; Cordyceps fosters mitochondrial bioenergetics and bolsters antioxidant capacity. Theoretically, these compounds could interact synergistically via a mitochondrial–methylation–neurotransmitter axis. For instance, an enhanced methylation status may modulate the expression of genes pertinent to mitochondrial biogenesis and dopamine receptor synthesis, whereas increased mitochondrial ATP production may support the biosynthesis of neurotransmitters and facilitate synaptic transmission. Collectively, these interactions may potentiate adaptations to physical exercise and enhance performance across a heterogeneous range of populations. Consequently, the objective of this comprehensive review is to construct and critically evaluate a conceptual, speculative model of mitochondrial–methylation–neurotransmitter interactions. This framework serves as an integrative mechanistic hypothesis rather than an empirically proven fact, outlining how betaine, tyrosine, and Cordyceps might theoretically exert synergistic influences on athletic performance. While we hypothesize that these three agents target overlapping nodes within a shared metabolic-epigenetic-neurological axis, it must be emphasized that direct co-supplementation data is currently absent. It is also important to emphasize that this review distinguishes between findings based on their level of evidence. Mechanistic pathways, particularly regarding specific molecular cascades, are largely derived from *in vitro* cell studies and animal models. Where human clinical data exists, these studies are highlighted to reflect translational relevance. Additionally, many cited studies utilize multi-ingredient formulations, which limits the ability to isolate the specific ergogenic effect of individual compounds like betaine, tyrosine, or Cordyceps.

## 2 Molecular mechanisms of the mitochondrial-methylation-neurotransmitter axis

The molecular basis of the mitochondrial-methylation-neurotransmitter crosstalk is fundamentally anchored in the energetic dependence of one-carbon metabolism. Mitochondria are responsible for supplying the adenosine triphosphate (ATP) required for the activation of methionine into S-adenosylmethionine (SAM), the universal methyl donor (16). This biochemical interdependency ensures that fluctuations in mitochondrial energy output directly dictate the cellular methylation potential (often represented by the SAM-to-SAH ratio), which subsequently governs downstream epigenetic and neurological processes.

In the context of exercise-induced adaptation, this crosstalk strongly influences epigenetic regulation and mitochondrial biogenesis. Metabolic stress during endurance exercise activates the AMP-activated protein kinase (AMPK) pathway. AMPK not only phosphorylates key regulators like PGC-1α but also interacts with the methylation machinery (17). Fluctuations in SAM availability modulate the activity of DNA methyltransferases (DNMTs), which can lead to targeted DNA hypomethylation at the PGC-1α promoter (10). This epigenetic modification facilitates enhanced transcription of PGC-1α and subsequent mitochondrial biogenesis, creating a positive feedback loop where augmented mitochondrial capacity further stabilizes one-carbon metabolism.

Concurrently, this metabolic-epigenetic axis directly interacts with neurotransmission, modulating central fatigue. The availability of SAM is a rate-limiting factor in both the synthesis and catabolism of key neurotransmitters. For instance, the conversion of norepinephrine to epinephrine by phenylethanolamine N-methyltransferase (PNMT), as well as the degradation of catecholamines by catechol-O-methyltransferase (COMT), are strictly SAM-dependent processes (18, 19). Therefore, shifts in one-carbon metabolism, driven by peripheral mitochondrial efficiency, may directly modulate central neurotransmitter tone, demonstrating a clear mechanistic bridge between cellular bioenergetics, epigenetic adaptations, and sustained nervous system output during prolonged exercise.

A critical distinction must be made between primary molecular mechanisms and secondary downstream consequences. For example, many studies report that these supplements “reduce fatigue” or “improve mitochondrial function.” However, a reduction in systemic fatigue is often a downstream consequence of improved cellular hydration (via betaine) or delayed central nervous system exhaustion (via tyrosine), rather than a direct enhancement of mitochondrial respiratory chain complexes. Similarly, improved antioxidant profiles observed with Cordyceps supplementation might not reflect a primary optimization of mitochondrial bioenergetics, but rather a secondary compensatory response to reduced oxidative damage. Differentiating these primary and secondary effects is crucial for understanding the true ergogenic limits of this synergistic model.

## 3 Effects of betaine supplementation with exercise on mitochondrial–methylation–neurotransmitter axis

Betaine constitutes a distinctive nutritional agent endowed with the capability to integrate metabolic, epigenetic, and neurochemical pathways, particularly when synergistically applied with exercise training. In contrast to numerous ergogenic supplements that focus on singular biological mechanisms, betaine operates at the convergence of one-carbon metabolism, osmotic homeostasis, redox equilibrium, and cellular bioenergetics. When amalgamated with exercise-induced signaling pathways, these attributes position betaine as a promising modulator of the mitochondrial–methylation–neurotransmitter axis, carrying significant implications for the enhancement of athletic performance, optimization of recovery processes, and bolstering of metabolic resilience.

In a double-blind human crossover trial, an investigation examined the impact of a two-week intervention involving betaine (3 g/day) in comparison to a placebo on the performance of 60 km cycling, gastrointestinal permeability, and alterations in plasma metabolite profiles among 21 non-elite male and female cyclists. A randomized, double-blind, placebo-controlled, crossover methodology was employed, consisting of two distinct two-week supplementation phases separated by a two-week washout period, culminating in a 60 km cycling time trial. Blood samples were acquired prior to and subsequent to the supplementation phases, as well as at various intervals following exercise, alongside five-hour urine collections. The outcome measures encompassed plasma intestinal fatty acid binding protein-1, biomarkers indicative of muscle damage (creatine kinase, myoglobin), serum cortisol levels, blood cell counts, and untargeted metabolomics analyses. The administration of betaine supplementation led to a statistically significant reduction in 60 km cycling time trial duration. Untargeted metabolomics revealed alterations in 214 plasma metabolites, characterized by significant increases in one-carbon intermediates, including betaine, dimethylglycine, sarcosine, and methionine, alongside elevations in S-adenosylhomocysteine (SAH), alpha-ketoglutarate, and 5′-methylthioadenosine. Concurrently, a distinct decrease in plasma carnitine and various acylcarnitines was observed, while markers of gastrointestinal permeability (such as I-FABP) and muscle damage (creatine kinase and myoglobin) remained unaltered (20) (Table 1). These shifts suggest that betaine substantially modulates one-carbon flux during post-exercise recovery. Building on these substrate alterations, energy metabolism strategies that promote the utilization of fatty acids while diminishing dependence on glucose have been proposed to enhance athletic performance. In a preclinical murine model, the aim of a study was to examine the impact of a multi-ingredient supplement (MIS) comprising valine, isoleucine, leucine, *β*-alanine, creatine, L-carnitine, quercetin, and betaine on endurance exercise in a murine model, employing metabolomics analysis. Serum metabolite profiling conducted through ultrahigh performance liquid chromatography-mass spectrometry demonstrated noteworthy modifications in lipid metabolism in MIS-administered mice in contrast to control subjects. In particular, levels of glycerophospholipids, glycerolipids, long-chain fatty acids, and derivatives of arachidonic acid were reduced, whereas concentrations of medium- to long-chain acylcarnitines, dimethylglycine, citrate, glycerol, creatine, and corticosterone were found to be elevated. These metabolic alterations were correlated with pathways associated with the citrate cycle, amino acid metabolism, and serotonergic signaling, indicating that MIS facilitates a shift in energy substrate utilization from glucose to lipids, which may contribute to the enhancement of endurance performance (21).

**Table 1:** Effects of betaine supplementation on exercise performance and metabolic outcomes.

Participants	Intervention	Exercise protocol	Main performance outcomes	Metabolic/biomarker findings	Ref
Twenty-one male and female non-elite cyclists	Betaine 3 g/day vs. placebo for 2 weeks	60 km cycling time trial; blood samples pre/post supplementation and 0 h, 1.5 h, 3 h, 24 h post-exercise; 5-h urine sugar permeability test	Significant reduction in time to complete 60 km (112.8 ± 2.3 vs. 114.2 ± 2.6 min; ES = 0.475; *p* = 0.042)	No differences in gut permeability (I-FABP, lactulose:mannitol), NLR, cortisol, myoglobin, CK. 214 metabolites showed treatment effects; ↑ betaine, DMG, sarcosine, methionine, SAH, α-ketoglutaramate, MTA; ↓ carnitine and acylcarnitines	Nieman et al. (20)
Mice	Multi-ingredient supplement (valine, isoleucine, leucine, β-alanine, creatine, L-carnitine, quercetin, betaine)	Endurance exercise protocol	Improved endurance performance (associated with shift in energy substrate use)	↓ glycerophospholipids, glycerolipids, long-chain fatty acids, arachidonic acid derivatives; ↑ medium–long-chain acylcarnitines; ↑ DMG, citrate, glycerol, creatine, corticosterone; pathway changes in TCA cycle, amino acid metabolism, serotonergic synapses	Haixia and Hui (21)
Eighteen recreationally trained males (25 ± 5 y)	Betaine 6 g/day vs. placebo for 14 days	One-leg press: BFR (20% 1RM, 80% occlusion) and high-load (70% 1RM) to failure; lactate and serum biomarkers measured	No significant difference in total repetitions between supplements	Lactate increased post-exercise (HL > BFR at 3 h); ↑ ΔIGF-1 in BET vs. PLA (p = 0.042); ↑ ΔHCY in HL vs. BFR; no effect on GH	Machek et al. (22)
Male Wistar albino rats	GAA (300 mg/kg), creatine (280 mg/kg), betaine (300 mg/kg), alone or combined (4 weeks post-HIIT)	4 weeks high-intensity interval training (HIIT)	No alteration in cardiac function; ↑ cardiomyocyte hypertrophy in HIIT + GAA (↑ cross-sectional area 27%; ↑ LV wall thickness 27%, *p* < 0.05)	↓ systemic and cardiac prooxidants (O₂^−^, H₂O₂, NO₂^−^, TBARS); ↑ glutathione and superoxide dismutase; strongest effect in GAA-only group	Prokic et al. (23)
Ten adolescent handball players (16 ± 1 yrs)	Betaine 2.5 g/day vs. placebo for 14 days (crossover)	High-intensity resistance exercise (leg press + bench press; five sets to fatigue at 80% 1RM)	↑ repetitions in leg press (35.8 vs. 24.8) and bench press (36.3 vs. 26.1), *p* < 0.001; large effect sizes	↓ post-exercise cortisol and lactate; ↑ total testosterone and T/C ratio (*p* < 0.001)	Arazi et al. (24)
Mice	7-day multi-ingredient formula (valine, isoleucine, leucine, β-alanine, creatine, L-carnitine, quercetin, betaine)	Exhaustive treadmill running test	↑ running duration (+14%) and distance (+20%)	↑ fatty acid oxidation; spared liver glycogen; ↑ p-AMPKα, p-ACC, CPT1B, CD36, GLUT4; ↑ antioxidant capacity	Chen et al. (25)
Twenty-three active college-age females	Betaine 2.4 g/day vs. placebo for 2 weeks (parallel design)	Graded cycling test + three 10 s sprints	↑ mean power output in final sprint (~3%, *p* = 0.02); ↓ RPE; ↑ fat-free mass (~3%)	No significant changes in substrate oxidation rates; improved body composition only in BET group	Waldman et al. (26)
Twenty-nine professional youth soccer players (15.45 ± 0.25 yrs)	Betaine 2 g/day (*n* = 14) vs. placebo (*n* = 15) for 14 weeks	Competitive soccer season (practices, conditioning, games); standardized workload	No significant effects on lean body mass or body fat	↑ Testosterone and ↑ T/C ratio at mid- and post-season in betaine group (*p* < 0.05); no effect on GH or IGF-1; greater increases in height and weight at 1-year follow-up in betaine group	Nobari et al. (27)
Twenty-nine trained subjects (≈4.2 training days/week)	Betaine vs. placebo for 6 weeks	CrossFit® training; 3-RM back squat, 2 km rowing test, Bergeron Beep Test	↑ back squat strength in betaine group (*p* = 0.04), but no between-group differences; no improvement in aerobic/anaerobic performance	No significant changes in body composition or cellular hydration; non-significant fat mass reduction in betaine group	Moro et al. (28)
Twenty-three untrained collegiate females (21.0 ± 1.4 yrs)	Betaine 2.5 g/day (*n* = 11) vs. placebo (*n* = 12) for 8 weeks	Resistance training (three sessions/week; lower–lower–upper split; sets to failure)	No significant interaction for strength (squat, bench press) or vertical jump; trend toward higher weekly training volume in betaine	Greater reductions in body fat % (−3.3%) and fat mass (−2.0 kg) vs. placebo (*p* < 0.05); no differential increase in lean mass	(29)
Twelve trained men (19.7 ± 1.23 yrs)	Betaine 1.25 g BID vs. placebo for 2 weeks (crossover)	Acute exercise session (AES) pre- and post-supplementation	No direct performance outcomes reported	↑ IGF-1 (*p* = 0.010); trend ↑ GH (*p* = 0.060); ↓ cortisol (*p* = 0.007); ↑ total Akt (*p* = 0.003); ↑ phosphorylation of Akt (Ser473) and p70S6k (Thr389); ↓ AMPK phosphorylation	Apicella et al. (30)
Resistance-trained men	Betaine 2.5 g/day vs. placebo for 14 days (crossover)	Bench press (10 sets to failure), muscular power and isometric force tests	↑ total repetitions (+6.5%) and ↑ total volume load in bench press (*p* < 0.05); no differences in other performance tests	↓ muscle StO₂ during exercise (*p* = 0.01); trend ↓ NOx (*p* = 0.06); lactate increase attenuated (NS); MDA unchanged	Trepanowski et al. (31)
Eleven active college-aged men (21.7 ± 5.1 yrs)	Betaine vs. placebo for 15 days (crossover)	Isokinetic chest press (concentric and eccentric force testing)	No changes in peak concentric or eccentric force	No differences in soreness; ↓ subjective fatigue in betaine vs. placebo	Hoffman et al. (32)
Untrained subjects	Betaine (2 g/day), Creatine (20 g/day), Betaine + Creatine (2 + 20 g/day), or placebo for 10 days	Strength and power testing (1-RM squat and bench press; muscle power; muscle PCr content assessment)	↑ Strength and power in CR and CR + BET groups; no differences between BET and placebo; no additive effect of BET + CR over CR alone	↑ Muscle PCr in CR and CR + BET; no change in PCr with BET alone; no body composition differences	del Favero et al. (33)
Ninety-five institutionalized older adults (83 ± 6 yrs.; 88% female)	Resistance training ± nutritional supplementation (Fortifit) for 6 months	Resistance training or cognitive training; plasma 1C metabolites and cardiometabolic markers measured at baseline, 3 and 6 months	Not performance-focused	↑ Choline (3 months); ↑ cysteine & methionine (6 months); higher baseline DMG and lower betaine associated with unfavorable cardiometabolic profile; ↑ choline and DMG linked to improved lipid metabolism in supplemented group	Gillies et al. (34)
Male C57BL/6 J mice with HFD-induced obesity	Betaine (1.5% w/v), treadmill exercise, or combined for 10 weeks	Treadmill exercise (15 m/min, 45 min/day, 6 days/week)	Exercise reduced body weight and adiposity; combined exercise + betaine normalized glucose tolerance vs. exercise alone	Combined treatment improved GTT and reduced insulin levels (↑ insulin sensitivity); normalized hepatic Mpc1; modest skeletal muscle fatty acid gene changes; limited hepatic lipid effects	Yu et al. (35)

In a human clinical trial involving resistance-trained males, another study evaluated the influence of a 14-day supplementation of betaine (6 g/day) on the performance of one-leg press exercises, lactate responses, and exercise-related biomarkers in a cohort of 18 recreationally trained male participants. Participants executed four standardized sets and two sets to failure under conditions of low-load blood flow restriction (LL-BFR; 20% 1RM at 80% arterial occlusion pressure) or high-load (HL; 70% 1RM). Lactate concentrations were quantified prior to, immediately following, 30 min after, and 3 h post-exercise, while serum levels of homocysteine (HCY), growth hormone (GH), and insulin-like growth factor-1 (IGF-1) were evaluated both pre- and 30 min post-exercise. No statistically significant differences in total repetitions were detected between the betaine and placebo conditions. The high-load condition resulted in a greater accumulation of lactate compared to the low-load blood flow restriction condition. Betaine supplementation significantly elevated post-exercise levels of IGF-1 in comparison to the placebo group, whereas levels of GH remained unchanged. HCY levels were observed to be higher in the high-load condition relative to those in the low-load blood flow restriction condition (22). In a preclinical animal model, a research endeavor examined the impact of guanidinoacetic acid (GAA), administered either singularly or in conjunction with betaine or creatine, on cardiac functionality, morphometric parameters, and redox equilibrium in male Wistar rats undergoing a regimen of high-intensity interval training (HIIT) for a duration of four weeks. The subjects were stratified into groups receiving HIIT, HIIT + GAA, HIIT + GAA + creatine, HIIT + GAA + betaine, or HIIT + GAA + creatine + betaine, with each group receiving daily oral supplementation. The supplementation of GAA resulted in a marked reduction of systemic and cardiac prooxidants, such as O₂^−^, H₂O₂, NO₂^−^, and TBARS, while concomitantly elevating levels of glutathione and superoxide dismutase. Although no treatment modalities produced alterations in cardiac functionality, the HIIT + GAA regimen precipitated cardiomyocyte hypertrophy, as evidenced by a 27% augmentation in cross-sectional area and left ventricular wall thickness (23).

An investigation sought to assess the impact of a 14-day regimen of betaine supplementation (2.5 g/day) on muscular endurance, plasma lactate levels, testosterone levels, cortisol levels, and the testosterone-to-cortisol (T/C) ratio among 10 adolescent handball athletes engaged in high-intensity resistance training. Employing a double-blind, crossover methodology with a 30-day washout period, participants executed leg press and bench press exercises to the point of volitional fatigue at 80% of their one-repetition maximum (1RM) both prior to and subsequent to the supplementation period. The betaine supplementation resulted in a statistically enhancement in the number of repetitions performed in both the leg press (35.8 ± 4.3 compared to 24.8 ± 3.6) and bench press (36.3 ± 2.6 compared to 26.1 ± 3.5), while concurrently decreasing post-exercise levels of cortisol and lactate, and increasing total testosterone levels along with the T/C ratio in comparison to the placebo condition (24). Exercise-induced fatigue encompasses the depletion of energy reserves, accumulation of metabolites, and the manifestation of oxidative stress, thereby substantiating a comprehensive, multi-targeted strategy for the enhancement of athletic performance. A study assessed a 7-day regimen of a MIS comprising valine, isoleucine, leucine, β-alanine, creatine, L-carnitine, quercetin, and betaine in murine models subjected to exhaustive treadmill exercise. Supplementation with MIS resulted in a 14% increase in running duration and a 20% enhancement in distance covered, signifying a postponement of fatigue onset and an augmentation of endurance capacity. From a mechanistic perspective, MIS facilitated an increase in fatty acid oxidation and conserved hepatic glycogen reserves through the modulation of various energy metabolism pathways, including p-AMPKα, p-ACC, CPT1B, CD36, and GLUT4, while concurrently enhancing antioxidant capacity (25). Another research endeavor examined the ramifications of a two-week regimen of betaine supplementation (2.4 g/day) on metabolic flexibility, body composition, and anaerobic performance among a cohort of 23 college-aged female participants. The subjects undertook a graded cycle ergometer assessment to evaluate fat and carbohydrate oxidation, subsequently engaging in three 10-s sprints. Employing a parallel-group design, participants were administered either betaine or a placebo, with evaluations conducted both prior to and following the supplementation period. Although no significant differences were detected in substrate oxidation rates, the betaine group uniquely exhibited an enhancement in fat-free mass (approximately 3%) and demonstrated improved performance metrics, including elevated mean power output during the final sprint and reduced perceived exertion in the concluding phase of the graded test (26).

The purpose of study was to determine the impact of a 14-week betaine supplementation regimen (2 g/day) on hormonal profiles, body composition metrics, and anthropometric variables in a cohort of 29 professional youth soccer athletes (15.5 ± 0.25 years) throughout a competitive season. The participants were stratified by playing position and subsequently randomized to receive either betaine (*n* = 14) or a placebo (*n* = 15), all while undergoing standardized training, conditioning, and competitive matches. Hormones including growth hormone, IGF-1, testosterone, and cortisol, alongside body composition parameters, stature, and weight, were evaluated at pre-, mid-, and post-season intervals, with an additional follow-up conducted 1 year later. The administration of betaine resulted in a statistically significant elevation in testosterone levels and an enhancement of the testosterone-to-cortisol (T/C) ratio at both mid- and post-season assessments, in comparison to baseline measurements, while also mitigating the decline in testosterone levels noted among the placebo group. No detrimental effects on growth, lean body mass, or adiposity were recorded (27). Another investigation explored the impact of a six-week regimen of betaine supplementation (BET) on body composition and muscular performance during CrossFit© training among 29 participants who were equitably matched for training status and body fat percentage. The subjects were randomly allocated to either the BET group (*n* = 14) or the placebo group (*n* = 15). Body composition and cellular hydration were evaluated through the utilization of skinfold measurements and bioelectrical impedance analysis, while muscular performance was assessed using a three-repetition maximum (3-RM) back squat, a two-kilometer rowing test, and the Bergeron Beep Test. The supplementation of betaine resulted in a statistically enhancement of muscle strength in the back squat; however, no notable differences were identified between the two groups. The fat mass exhibited a tendency to decrease within the BET group, yet there were no substantial alterations in overall body composition or cellular hydration. In summary, short-term betaine supplementation appears to facilitate strength developments but does not significantly augment aerobic or anaerobic performance in individuals engaged in CrossFit© training (28).

An investigation systematically assessed the impact of an eight-week regimen of resistance training in conjunction with betaine supplementation (2.5 g/day) on body composition and muscular performance in a cohort of 23 untrained young women. Subsequent to a 1-week acclimatization period, participants were equitably matched and randomly allocated to either the betaine group (*n* = 11) or the placebo group (*n* = 12), engaging in three weekly sessions of multi-faceted resistance training exercises. The assessments conducted prior to and following the training intervention encompassed measurements of lean mass, body fat, rectus femoris thickness, vertical jump performance, and one-repetition maximum (1RM) for both squat and bench press exercises. Although both cohorts demonstrated improvements in lean mass, muscle thickness, jump performance, and overall strength, betaine supplementation was found to significantly augment reductions in body fat percentage (−3.3% vs. -1.7%) and fat mass (−2.0 kg vs. -0.8 kg) in comparison to the placebo group (29). The objective of a work was to examine the ramifications of a 2-week regimen of betaine supplementation (1.25 g administered bi-daily) on circulating hormones and signaling pathways in skeletal muscle among 12 physically trained male participants, employing a double-blind, crossover methodology accompanied by a 2-week washout period. Subsequent to acute exercise sessions, measurements of circulating growth hormone (GH), insulin-like growth factor 1 (IGF-1), cortisol, and insulin were conducted, while vastus lateralis biopsies were utilized to evaluate the signaling of Akt, p70 S6 kinase, and AMPK. The administration of betaine resulted in an elevation of IGF-1 levels and resting total muscle Akt, enhanced the phosphorylation of Akt at Ser473 and p70 S6k at Thr389, and diminished cortisol levels when contrasted with the placebo group; however, insulin levels and AMPK phosphorylation remained unchanged (30).

A research assessed the impact of a 14-day regimen of betaine supplementation (2.5 g/day in Gatorade®) on exercise efficacy, muscle oxygen saturation, and biochemical indicators in resistance-trained males, employing a double-blind, crossover methodology with a 21-day washout period. Participants engaged in assessments of upper- and lower-body power, isometric strength, and a 10-set bench press to failure. Muscle tissue oxygen saturation (StO₂) was quantified utilizing near-infrared spectroscopy, while blood lactate, nitrate/nitrite (NOx), and malondialdehyde (MDA) were subjected to analysis. Betaine supplementation significantly augmented total repetitions and volume load in the 10-set bench press (~6.5%), whereas other performance metrics remained unaffected. Post-exercise lactate levels exhibited a tendency to decrease, NOx levels diminished following the intervention, and StO₂ showed a more pronounced decline with betaine, indicating an enhancement in muscle oxygen utilization (31). Another study assessed the implications of a 15-day betaine supplementation regimen on peak concentric (CON) and eccentric (ECC) force, fatigue levels, and muscle soreness during an isokinetic chest press exercise among 11 physically active male college students (21.7 ± 5.1 years) employing a double-blind, crossover methodology. Participants underwent pre- and post-intervention assessments during the supplementation phase and subsequent to a 4-week washout interval, during which they alternated conditions. The administration of betaine did not yield statistically significant alterations in maximum CON or ECC force relative to the placebo condition. Although no significant differences in subjective muscle soreness were detected, betaine supplementation was found to considerably mitigate exercise-induced fatigue (32). A double-blind, randomized, placebo-controlled investigation examined the effects of a 10-day regimen of betaine (2 g/day), creatine (20 g/day), or their synergistic combination on muscle phosphorylcreatine (PCr) levels, strength, power, and body composition in untrained individuals. The evaluation of muscle strength and power was conducted through squat and bench press exercises. It was found that creatine and the combination of betaine and creatine enhanced muscle PCr levels, power output, and one-repetition maximum (1-RM) strength in comparison to the placebo group, whereas betaine administered alone did not exhibit any effects on PCr, strength, or power. Furthermore, body composition remained consistent across all experimental groups (33).

Another research examined the impact of resistance training in conjunction with nutritional supplementation on the concentration of one-carbon (1C) metabolites and cardiometabolic health among a cohort of 95 institutionalized elderly individuals (mean age 83 ± 6 years, with 88.4% being female) over a duration of 6 months. Participants were randomly assigned to engage in resistance training either with or without the nutritional supplement (Fortifit), or to partake in cognitive training as a control group. Plasma levels of 1C metabolites and various cardiometabolic markers were assessed at the outset of the study, as well as at the 3- and 6-month intervals. Notably, across the different interventions, choline levels exhibited an increase at the 3-month mark, while the concentrations of cysteine and methionine remained significantly elevated at the 6-month follow-up. Additionally, higher baseline levels of dimethylglycine and lower levels of betaine were found to be associated with poorer cardiometabolic profiles, whereas increases in choline and dimethylglycine were positively correlated with enhanced lipid metabolism among participants receiving supplementation (34). Another work determined the impact of betaine supplementation, treadmill exercise, and their synergistic effect on obesity induced by a high-fat diet (HFD) in male C57BL/6 J mice. Following a 15-week period on HFD, the mice were administered betaine (1.5% w/v), engaged in exercise (15 m/min, 45 min/day, 6 days/week), or participated in both interventions for a duration of 10 weeks. Exercise as a singular intervention resulted in a reduction in body weight, adiposity, and a modest enhancement in glucose tolerance, while betaine administered in isolation exhibited negligible effects. The concurrent application of betaine and exercise yielded superior outcomes compared to exercise alone, effectively normalizing glucose tolerance and decreasing insulin levels, indicative of improved insulin sensitivity. Gene expression related to fatty acid metabolism in skeletal muscle demonstrated slight enhancements, whereas the hepatic responses appeared to be limited (35). Betaine demonstrates a diverse array of molecular effects that enhance physical performance, body composition, and metabolic health. From a mechanistic perspective, betaine functions as a methyl donor within one-carbon metabolism, elevating levels of SAM and dimethylglycine, which are pivotal in supporting the remethylation of homocysteine, lipid metabolism, and the regulation of cardiometabolic health. It modulates the utilization of energy substrates by facilitating fatty acid oxidation, preserving glycogen reserves, and modifying acylcarnitine profiles, thereby contributing to prolonged fatigue resistance and augmented endurance. Furthermore, betaine exerts influence on anabolic signaling pathways through the upregulation of IGF-1, the enhanced phosphorylation of Akt and p70 S6 kinase, and the attenuation of cortisol levels, thereby favoring protein synthesis and muscle hypertrophy. When combined with amino acids or creatine, it can synergistically enhance mitochondrial function, bolster antioxidant capacity, and modulate the phospho-AMPKα/ACC signaling pathways. Additionally, betaine affects muscle hydration, metabolic flexibility, and substrate partitioning, with responses that are contingent upon sex, age, and exercise modalities, thereby underscoring its potential utility in personalized nutrition and exercise strategies.

Critical analysis of betaine evidence divergence.

A critical comparison of the available evidence reveals clear boundaries to betaine’s ergogenic efficacy. Human trials operating under high-intensity, fixed-workload resistance or cycling protocols frequently report positive outcomes regarding volume load (+6.5% in bench press) and time-trial performance. Conversely, when betaine is applied to highly chaotic training modalities such as CrossFit© or evaluated as a standalone agent for maximal force output, null findings predominate. This discrepancy indicates that betaine functions primarily as an osmolyte and metabolic stabilizer that delays exhaustion during prolonged, predictable metabolic strain, rather than acting as an acute neuromuscular potentiation agent. Furthermore, its efficacy is severely obscured in multi-ingredient formulas, where the precise contribution of betaine cannot be isolated from co-administered agents like creatine or beta-alanine.

## 4 Effects of tyrosine supplementation with exercise on mitochondrial–methylation–neurotransmitter axis

Tyrosine, recognized as a conditionally essential aromatic amino acid, assumes a pivotal biochemical role at the intersection of neurotransmitter biosynthesis, stress response, and metabolic homeostasis. As the primary precursor for the neurotransmitters dopamine, norepinephrine, and epinephrine, the bioavailability of tyrosine can significantly affect motivational drives, motor coordination, and cognitive resilience in the context of physical stressors. When integrated with physical exercise—a potent influencer of mitochondrial biogenesis and epigenetic modifications—tyrosine supplementation has the potential to yield synergistic impacts across the mitochondrial–methylation–neurotransmitter continuum, transcending its traditional function in catecholamine production.

In a human clinical crossover study under environmental stress, an investigation examined the implications of tyrosine supplementation (150 mg/kg) on both physical and cognitive performance during extended periods of high-intensity intermittent exercise conducted in elevated thermal conditions (32 °C, 50% relative humidity) involving eight trained male soccer athletes. Participants engaged in a 92-min cycling sprint protocol that incorporated soccer-specific decision-making tasks under both tyrosine and placebo conditions within a counterbalanced design framework. The outcomes assessed encompassed peak power output, decision-making accuracy, cognitive appraisal, and affective states. No statistically significant differences or interactions between condition and time were detected between the tyrosine and placebo groups for any of the measured variables. These results suggest that acute tyrosine supplementation does not effectively enhance self-paced sprint performance, decision-making processes, or perceptual strain in hot environmental conditions (36) (Table 2). In a human trial evaluating a multi-ingredient pre-workout supplement (MIPS), another research investigated the comparative efficacy of a multi-ingredient pre-workout supplement (MIPS; comprising beta-alanine, L-citrulline malate, arginine α-ketoglutarate, L-taurine, L-tyrosine, and caffeine) against an equivalent dosage of anhydrous caffeine in relation to bench press strength endurance and psychological parameters. Fifteen resistance-trained male subjects (83.9 ± 8.9 kg; 5.6 ± 3.4 years of training experience) executed 10-repetition maximum bench press assessments within a randomized, double-blind, crossover framework. Pre- and post-exercise evaluations encompassed the Feeling Scale (FS), Felt Arousal Scale (FAS), and session rating of perceived exertion (sRPE). The volume of bench press repetitions was significantly greater following the administration of caffeine alone in comparison to MIPS, whereas no significant differences were detected in psychological assessments (37).

**Table 2:** Effects of tyrosine supplementation on performance, cognition, metabolic outcomes, and energy expenditure.

Participants	Intervention	Exercise protocol	Main performance outcomes	Metabolic/biomarker findings	Ref
Eight trained male soccer players	Tyrosine (150 mg·kg^−1^) vs. placebo	92-min high-intensity intermittent cycling sprint protocol with soccer-specific decision-making tasks in 32 °C (50% RH)	No differences in peak power output or decision-making accuracy	No differences in cognitive appraisal or affective states; no condition × time effects	Donnan et al. (36)
Fifteen resistance-trained males	Multi-ingredient pre-workout supplement (beta-alanine, citrulline malate, arginine AKG, taurine, tyrosine, caffeine) vs. caffeine alone	Bench press strength endurance (10RM-based protocol); psychological assessments (FS, FAS, sRPE)	No added psychological benefit of MIPS compared to caffeine	MIPS was less ergogenic and less cost-effective than caffeine alone for bench press endurance	Kruszewski et al. (37)
Twelve college-aged males (22.8 ± 2.4 yrs)	Multi-ingredient pre-workout (caffeine, green tea, yohimbe, capsicum, coleus, L-carnitine, beta-alanine, tyrosine) vs. caffeine (6 mg·kg^−1^) vs. placebo	60-min low-intensity treadmill exercise	↑ VO₂, VCO₂, VE vs. placebo; no changes in substrate oxidation; ↑ alertness and focus; ↓ fatigue with pre-workout vs. placebo	Multi-ingredient pre-workout increased energy expenditure similarly to caffeine; improved alertness and reduced fatigue but no added substrate oxidation benefits	Lutsch et al. (38)
Twenty male collegiate athletes (20.5 ± 1.4 y)	Proprietary supplement (caffeine + theanine + tyrosine) vs. placebo (acute ingestion)	Two rounds of exercise with mental/physical performance testing (Makoto Arena; static and dynamic reactive tasks)	↑ Movement accuracy (static & dynamic); ↑ targets hit and ↓ hit time (dynamic only); No change in exercise performance or subjective variables	No metabolic markers reported	Zaragoza et al. (39)
Thirty-two male Wistar rats	L-tyrosine (500 mg/kg/day), exercise, exercise + L-tyrosine, control (10 weeks)	Moderate treadmill running (5 days/week for 10 weeks); penicillin-induced epileptiform activity recorded post-intervention	↓ Epileptiform spike frequency in exercise, L-tyrosine, and combined groups; no change in amplitude or latency	Electrocorticogram (ECoG): reduced spike frequency; no change in amplitude or latency	Kayacan et al. (40)
Zebrafish and mouse models of ACTA1 nemaline myopathy	L-tyrosine supplementation (up to 2% diet in mice; 10 μM in zebrafish)	Zebrafish: swimming assessment (24 h–6 d post-fertilization); mice: voluntary exercise, rotarod, muscle testing	No improvement in swimming distance, voluntary activity, rotarod performance, muscle strength, or mechanical properties	↑ Serum (+57) and quadriceps muscle (+45%) L-tyrosine levels; no improvement in muscle metabolism or atrophy markers	Messineo et al. (41)
Twenty-five healthy, recreationally active males and females	Acute pre-workout supplement (PWS) with or without p-synephrine vs. placebo (PLA); ingredients: beta-alanine 3 g, creatine nitrate 2 g, arginine alpha-ketoglutarate 2 g, N-acetyl-L-tyrosine 300 mg, caffeine 284 mg, *Mucuna pruriens* extract 15 mg L-Dopa, vitamins B/C, maltodextrin	Bench and leg press (2×10 @ 70% 1RM + 1 set to failure); Wingate anaerobic sprint; cognitive testing (Stroop)	↑ Resting energy expenditure (REE); ↑ perceived readiness, vigor, energy; ↑ cognitive function (1–2 h post-ingestion); no change in muscular endurance or Wingate sprint performance	Heart rate, blood pressure, ECG, general blood panels – no clinically significant changes	Jung et al. (42)
Eighty resistance-trained males	Eight-week supplementation of PWS or PWS + p-synephrine vs. placebo	Standardized resistance training; assessed at 0, 4, and 8 weeks; bench/leg press 1RM, Wingate, cognitive testing (Stroop), body composition (DXA)	Some improvements in cognitive function and 1RM at 4 weeks for PWS and PWS + S; no additive benefit of synephrine; responses similar to PLA at 8 weeks	Blood chemistry panels, HR, BP – no adverse changes; no additive metabolic effects from synephrine	Jung et al. (43)
Study 1: 21; Study 2: 8 military participants	Tyrosine (75 or 150 mg/kg) vs. placebo (sugar-free drink)	Study 2: 60-min walk + 2.4 km time trial carrying 25 kg backpack in heat (40 °C, 30% RH)	Tyrosine did not improve cognitive function or physical performance under heat stress; no effect on time trial completion	Serum tyrosine concentration ↑ after supplementation; no physiological or metabolic changes	Coull et al. (44)
Seven male endurance-trained volunteers (unacclimated to heat)	Acute oral tyrosine 150 mg/kg vs. placebo (whey powder)	60 min cycling at 57% ± 4% VO₂peak + simulated cycling time trial (target work 393.1 ± 39.8 kJ) at 30 °C, 60% RH	No change in time-trial power output or completion time; RPE, thermal sensation, core and skin temperature, HR unchanged	Plasma tyrosine + phenylalanine to competing amino acids ratio ↑ 2.5-fold with TYR; remained elevated during exercise; PLA declined	Tumilty et al. (45)
Eight healthy male volunteers (moderately trained)	Acute oral tyrosine 150 mg/kg vs. placebo (flavored sugar-free drink)	Cycling to exhaustion at 68 ± 5% VO₂peak at 30 °C, 60% RH	↑ Endurance time with TYR (80.3 ± 19.7 min vs. 69.2 ± 14.0 min, *p* < 0.01); RPE, thermal sensation, HR, core and skin temperature unchanged	Pre-exercise plasma tyrosine:large neutral amino acids ratio ↑ 2.9-fold in TYR; no change in PLA	Tumilty et al. (46)

The aim of study was to assess the immediate impacts of a multi-ingredient pre-workout supplement (comprising caffeine, green tea extract, Yohimbe, capsicum, coleus extract, L-carnitine, beta-alanine, and tyrosine) in comparison to a dosage of 6 mg/kg caffeine and a placebo on variables such as energy expenditure, substrate utilization, gas exchange, and psychological responses during a 60-min session of low-intensity treadmill exercise among 12 male participants (mean age 22.8 ± 2.4 years). Both the multi-ingredient pre-workout supplement and caffeine demonstrated a statistically significant increase in energy expenditure, VO₂, VCO₂, and ventilation when juxtaposed with the placebo condition. No significant differences were detected concerning fat or carbohydrate oxidation or the respiratory exchange ratio. Furthermore, the multi-ingredient pre-workout supplement was found to potentially enhance alertness and concentration while simultaneously diminishing perceived fatigue throughout the exercise session (38). A randomized, double-blind, placebo-controlled crossover investigation sought to determine if a supplement comprising caffeine, theamine, and tyrosine (SUP) augments cognitive and physical performance among a cohort of 20 male collegiate athletes (20.5 ± 1.4 years). The participants engaged in evaluations of movement precision and their responses to auditory and visual stimuli in both static and dynamic conditions prior to and subsequent to the ingestion of either SUP or a placebo, in addition to following two exercise sessions. The findings indicated that SUP may enhance movement accuracy in both static and dynamic assessments, and during the dynamic evaluations, it resulted in an increased number of targets successfully hit while concurrently decreasing average hit time in comparison to the placebo (*p* < 0.05). There were no significant differences noted in subjective evaluations or performance during the exercise rounds (39).

An investigation aimed to elucidate the impact of treadmill exercise and L-tyrosine supplementation on epileptiform activity in a cohort of 32 male Wistar rats. The subjects were stratified into distinct groups: control, exercise, L-tyrosine (500 mg/kg/day), and a combined exercise + L-tyrosine group, with interventions administered five times weekly over a duration of 10 weeks. Epileptiform activity was elicited through the administration of penicillin into the cerebral cortex, and subsequent electrocorticogram (ECoG) recordings were meticulously analyzed for variations in spike frequency and amplitude. In comparison to the control group, all treatment cohorts exhibited a statistically significant decrease in spike frequency, whereas no discernible differences were noted in spike amplitude or latency (40). In a preclinical rodent study, another work determined the impact of L-tyrosine supplementation on skeletal muscle functionality across three distinct animal models of nemaline myopathy (NM) attributed to dominant ACTA1 mutations. Safe dosing concentrations were established for both zebrafish and murine subjects. NM TgACTA1D286G-eGFP zebrafish that received a treatment of 10 μM L-tyrosine exhibited no enhancement in swimming performance. NM TgACTA1D286G mice provided with a diet supplemented with 2% L-tyrosine demonstrated notable elevations in serum (57%) and muscle (45%) L-tyrosine concentrations at the 6–7-week mark; however, metrics such as voluntary exercise, body weight, and rotarod performance remained unchanged. Furthermore, a 4-week supplementation regimen in 6–7 month-old TgACTA1D286G and KIActa1H40Y mice did not yield improvements in muscle mechanical properties, energy metabolism, or indicators of atrophy (41). The aim of study was to examine the immediate impacts of a multi-ingredient pre-workout supplement (PWS), both with and without the inclusion of 20 mg p-synephrine, on cognitive functioning, perceptions of readiness, exercise performance, and safety among 25 recreationally active adults. Participants consumed either a placebo, PWS, or PWS + S prior to engaging in exercise assessments, which comprised bench and leg press sets alongside a Wingate anaerobic sprint. Measurements of heart rate, blood pressure, electrocardiogram (ECG), and various blood biomarkers indicated no clinically significant alterations. Both PWS and PWS + S were associated with an increase in resting energy expenditure and an enhancement in self-reported vigor, optimism, and cognitive function when contrasted with the placebo group, while muscular performance metrics remained unchanged. The inclusion of p-synephrine yielded negligible additional advantages. The supplement was tolerated well by participants, suggesting that PWS predominantly augments cognitive and perceptual readiness rather than providing acute improvements in strength or anaerobic performance (42).

A study demonstrated the ramifications of an 8-week pre-workout supplement (PWS) with and without 20 mg of synephrine (PWS + S) on the physiological adaptations to training in a cohort of 80 resistance-trained males. The participants were systematically allocated to receive either a placebo, PWS, or PWS + S while engaging in a regimented resistance training protocol. Evaluative measures encompassed body composition, one-repetition maximum (1RM) strength, Wingate anaerobic capacity, cognitive performance, and various blood biomarkers at baseline, 4 weeks, and 8 weeks. Collectively, no statistically significant distinctions were detected among the groups concerning body composition, strength, anaerobic performance, or biochemical profiles. Noteworthy enhancements in cognitive performance and 1RM strength were observed at the 4-week mark for both PWS and PWS + S in comparison to the placebo; however, these enhancements were not maintained at the 8-week evaluation. The inclusion of synephrine did not confer any supplementary benefits, and the supplementation was generally well-accepted by the participants (43). Another investigation sought to elucidate the ramifications of tyrosine (TYR) supplementation on serum TYR concentrations, cognitive functioning, and physical performance in the context of thermal stress. In Study 1, a comparative analysis was conducted between a single administration (150 mg/kg) and a double administration (2 × 150 mg/kg), revealing that a solitary dose was adequate to augment serum TYR levels. Study 2 implemented this optimal dosage within a military-style load carriage protocol (60 min of ambulation coupled with a 2.4-kilometer time trial while carrying a 25-kilogram backpack at 40 °C and 30% relative humidity) utilizing a double-blind crossover framework. Although thermal stress adversely affected cognitive functioning, TYR supplementation failed to mitigate these impairments. No significant differences were detected in physiological responses or time trial performance between the TYR supplementation and placebo conditions. Consequently, despite the elevation of serum TYR levels, supplementation did not facilitate enhancements in cognitive or physical performance under heat stress conditions (44).

A study aimed to determine whether acute oral administration of tyrosine (TYR, 150 mg/kg) enhances self-paced endurance exercise performance in elevated temperature conditions. Seven male individuals with endurance training experience participated in two 60-min cycling trials at an intensity of 57% ± 4% VO₂peak, followed by a time trial, after consuming either TYR or a placebo (whey). The supplementation of TYR resulted in a significant elevation in the plasma ratio of tyrosine plus phenylalanine relative to competing large neutral amino acids (approximately 2.5-fold), thereby sustaining heightened availability of brain precursors throughout the duration of exercise. Notwithstanding this finding, no significant differences were observed between the TYR and placebo conditions in terms of time-trial performance, power output, ratings of perceived exertion, core or skin temperature, heart rate, or thermal sensation (45). A randomized, double-blind crossover investigation aimed to ascertain whether acute supplementation with tyrosine (TYR, 150 mg/kg) enhances endurance exercise tolerance in elevated thermal conditions by augmenting cerebral dopamine availability. Eight moderately conditioned male participants engaged in constant-load cycling until volitional fatigue (68 ± 5% VO₂peak) at a temperature of 30 °C and relative humidity of 60% after the administration of either TYR or a placebo. The pre-exercise plasma concentration of tyrosine, in relation to competing large neutral amino acids, exhibited a notable 2.9-fold increase with TYR, whereas no such effect was observed with the placebo. The administration of TYR significantly prolonged the duration of cycling until exhaustion (80.3 ± 19.7 min versus 69.2 ± 14.0 min, *p* < 0.01), without eliciting alterations in core or skin temperature, heart rate, perceived exertion, or thermal sensation (46). Acute supplementation with tyrosine is associated with an increase in plasma tyrosine-to-large neutral amino acid ratios, thereby facilitating the uptake of this amino acid within the central nervous system and promoting the synthesis of catecholamines, most notably dopamine and norepinephrine. Enhanced concentrations of brain catecholamines play a pivotal role in modulating neural signaling across both motor and cognitive circuits, which may contribute to the postponement of central fatigue and the enhancement of exercise tolerance under conditions of heat or stress. Multi-ingredient preworkout formulations, which encompass tyrosine, caffeine, beta-alanine, L-carnitine, and various other bioactive compounds, have been shown to elevate energy expenditure, VO₂, VCO₂, and levels of alertness through synergistic activation of adrenergic and neuromodulatory pathways. The combination of tyrosine with caffeine and theanine may improve movement accuracy by influencing synaptic neurotransmission, while not affecting the perception of exertion. Nevertheless, the effects of tyrosine are contingent upon contextual factors; significant enhancements are noted during constant-load endurance or stress-induced protocols, whereas self-paced, intermittent, or high-intensity exercise typically yields negligible benefits. While animal studies provide essential mechanistic data regarding neurotransmitter signaling in pathological states, clinical investigations in humans are essential to confirm the practical application of tyrosine as a standalone ergogenic aid under varying physiological stressors.

In summary, the actions of tyrosine and multi-ingredient supplements predominantly operate through the modulation of central catecholamines and neural excitability, with secondary implications for metabolic and psychophysiological responses.

## 5 Critical analysis of tyrosine evidence divergence

The ergogenic utility of tyrosine is strictly context-dependent, exhibiting a profound divergence between fixed-load and self-paced exercise tasks. Standalone tyrosine administration successfully prolongs time-to-exhaustion during constant-load cycling in environmental heat (+11 min), driven by augmented central dopamine availability. However, when athletes are subjected to self-paced time trials or intermittent sprint protocols, tyrosine consistently fails to yield performance gains. This suggests that while tyrosine provides a neurochemical buffer against central fatigue under passive thermal or physiological distress, it cannot override conscious pacing strategies or primary peripheral muscular limitations during self-regulated, high-intensity performance.

## 6 Cordyceps: phytochemical composition and bioactive compounds

The genus Cordyceps encompasses more than 400 species of parasitic fungi that are predominantly located in humid temperate and tropical forest ecosystems, where they engage in parasitism of insects and other arthropods. Notably, Cordyceps sinensis and Cordyceps militaris have been the subjects of extensive scholarly investigation owing to their historical applications in traditional medicine and their prospective therapeutic implications. *C. sinensis* is naturally found in the high-altitude regions of the Tibetan Plateau and the Himalayas, where it specifically parasitizes caterpillar larvae, thereby establishing its distinctive ecological niche and intricate life cycle (47, 48). Nevertheless, its inherent scarcity in nature and the complexities associated with its harvesting have redirected research and commercial focus towards *C. militaris*, which can be cultivated on agricultural substrates in regulated environments, thereby presenting a sustainable and more accessible alternative (49, 50). The pharmacological properties of Cordyceps species are predominantly ascribed to a wide variety of bioactive compounds, among which the most prominent include cordycepin, adenosine, polysaccharides, sterols, nucleosides, and an array of peptides.

Cordycepin is recognized as a pivotal metabolite exhibiting diverse biological functions, encompassing anti-inflammatory, antioxidant, and modulation of energy metabolism. Demonstrating structural resemblance to adenosine, cordycepin possesses the ability to disrupt nucleic acid biosynthesis and cellular signaling pathways, which may elucidate its potential to augment mitochondrial functionality and ATP synthesis within muscle cells (51, 52). Polysaccharides derived from Cordyceps manifest immunomodulatory characteristics and function as antioxidants by neutralizing free radicals produced during oxidative stress. These intricate carbohydrates may additionally play a role in enhancing exercise endurance and recovery through the facilitation of systemic immune responses (53, 54). Furthermore, adenosine and its analogs are acknowledged for their impact on vascular regulation and energy metabolism, both of which are essential for optimal oxygen delivery and utilization throughout physical activity (55, 56). Notwithstanding these encouraging bioactivities, the standardization of Cordyceps supplements poses a considerable challenge within both investigational and commercial realms. Variability in the origins of species, cultivation environments, extraction methodologies, and processing techniques results in significant heterogeneity in the phytochemical profiles of commercially available products. This inconsistency complicates the interpretation of clinical findings and restricts reproducibility across various research investigations. Standardization initiatives frequently emphasize the quantification of specific marker compounds, such as cordycepin or total polysaccharide content; however, there are no universally recognized protocols, and the bioavailability of these compounds in human subjects remains inadequately understood (57, 58). Dietary supplements are offered in an array of forms, encompassing powders, capsules, tinctures, and extracts, each exhibiting varying concentrations and stability of bioactive compounds. The selection of formulation may influence absorption kinetics and, consequently, the efficacy of Cordyceps-based interventions in athletic populations (49, 59). Hence, the establishment of rigorous quality control measures and the development of optimized delivery systems are imperative for the enhancement of the ergogenic potential of Cordyceps supplements. Noteworthy, a major critical limitation in the current literature is the inappropriate grouping of different Cordyceps interventions. Cordyceps sinensis, Cordyceps militaris, lab-grown mycelial extracts, and isolated cordycepin are distinct biological entities and cannot be treated as equivalent interventions. If the active compound concentration varies dramatically between commercial products, it raises several unanswered scientific questions. Which specific compound is responsible for the observed effects? What exact dose is required to elicit a physiological response in humans? Currently, the literature cannot answer whether over-the-counter commercial supplements are equivalent to the highly controlled extracts used in preclinical trials. Until standardized dosing protocols for specific isolated compounds like cordycepin are established in human models, generalized claims regarding Cordyceps supplementation must be interpreted with extreme caution.

## 7 Mitochondrial energy regulation and anti-fatigue effects of Cordyceps

Mitochondrial dysfunction, along with compromised energy metabolism, represents significant factors contributing to muscle fatigue and diminished athletic performance during periods of intense or extended physical exertion. To rigorously evaluate these effects, a clear distinction must be made between distinct biological processes that are frequently conflated in the literature:

- Mitochondrial biogenesis refers to the cellular synthesis of new mitochondria, a process primarily mediated by peroxisome proliferator-activated receptor-gamma coactivator 1-alpha (PGC-1α).
- Mitochondrial respiration involves the flux of electrons through the electron transport chain coupled to oxidative phosphorylation for ATP production.
- Mitochondrial efficiency explicitly relates to bioenergetic coupling, or the ratio of ATP synthesized per unit of oxygen consumed.
- Substrate oxidation entails the catabolic breakdown of carbohydrates or lipids to supply reducing equivalents to the tricarboxylic acid (TCA) cycle.
- Antioxidant defense encompasses the enzymatic and non-enzymatic neutralization of reactive oxygen species (ROS) generated as metabolic byproducts of these oxidative processes.

While current literature suggests *Cordyceps* primarily modulates antioxidant defense mechanisms and potentially upregulates mitochondrial biogenesis in preclinical models, robust clinical evidence directly demonstrating an increase in human mitochondrial respiration or net metabolic efficiency remains scarce.

**Figure 1:**
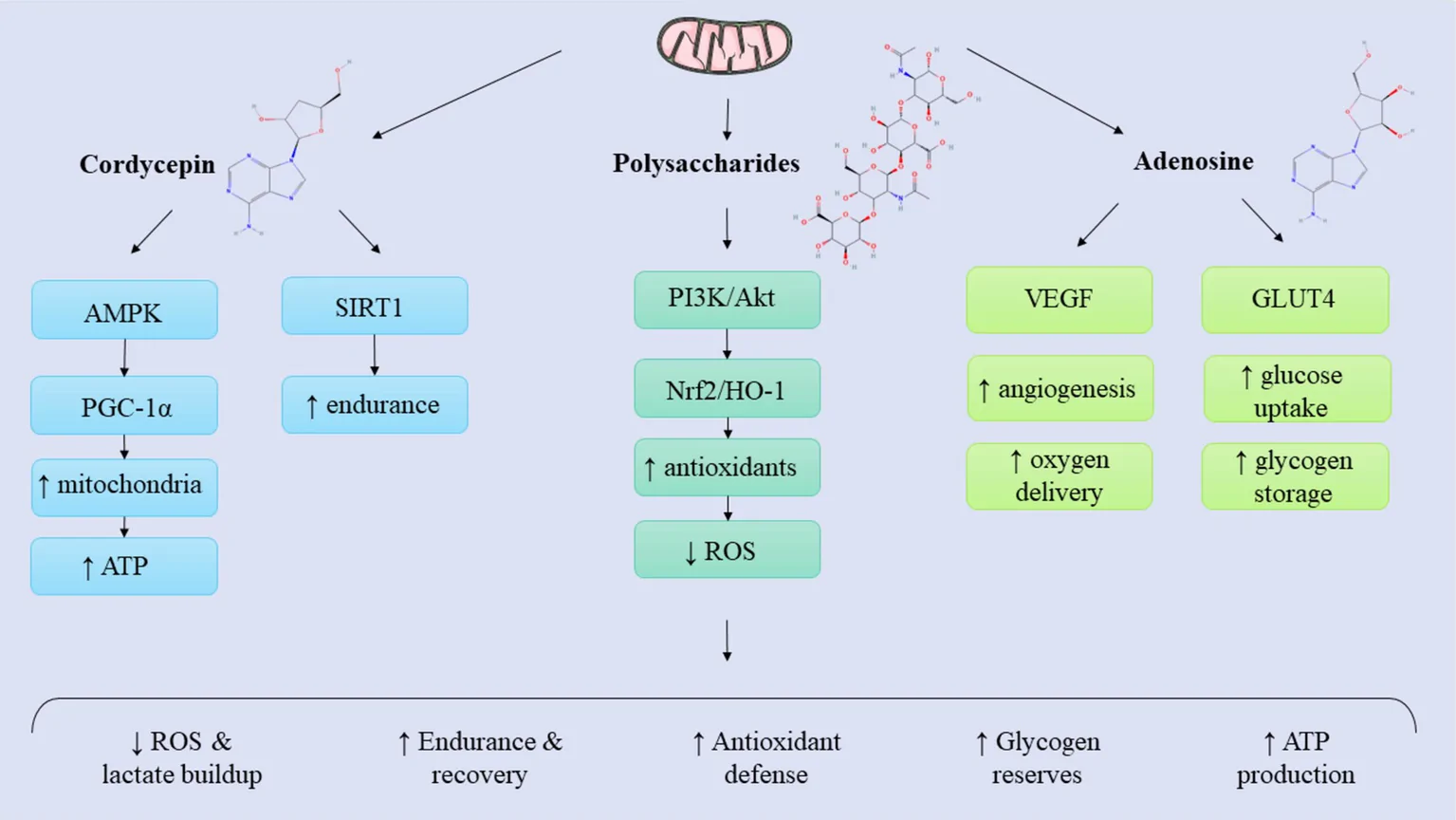
Schematic overview of Cordyceps-derived bioactives and their molecular targets. Cordycepin activates AMPK/PGC-1α and SIRT1 pathways, enhancing mitochondrial biogenesis and ATP synthesis. Polysaccharides stimulate PI3K/Akt-Nrf2/HO-1 signaling to boost antioxidant defense, while adenosine analogs increase VEGF-mediated angiogenesis and GLUT4-dependent glucose uptake. AMPK, AMP-activated protein kinase; PGC-1α, peroxisome proliferator-activated receptor *γ* coactivator-1α; SIRT1, sirtuin 1; Nrf2, nuclear factor erythroid 2–related factor 2; HO-1, heme oxygenase-1; VEGF, vascular endothelial growth factor; GLUT4, glucose transporter-4.

This section elucidates the molecular mechanisms through which Cordyceps enhances mitochondrial efficacy and alleviates exercise-induced fatigue, thereby providing a scientific foundation for its application as an ergogenic aid (Figure 1). A research endeavor was conducted to elucidate the anti-fatigue properties and the underlying mechanisms of cordycepin, a bioactive compound derived from Cordyceps militaris, utilizing a forced exercise mouse model. Excessive physical activity precipitates the onset of physical fatigue, oxidative stress, and cognitive deterioration. The administration of cordycepin resulted in enhanced exercise endurance, elevated glycogen reserves, and a reduction in serum markers associated with fatigue. Furthermore, it augmented the activity of antioxidant enzymes while concurrently diminishing oxidative damage. Behavioral assessments revealed that cordycepin alleviated cognitive impairments induced by exercise through the reduction of oxidative stress and the modulation of neurotransmitter levels. At the mechanistic level, cordycepin influenced the Keap1/Nrf2/HO-1 signaling pathway and elevated the expression of BDNF, thereby contributing to its neuroprotective effects. These findings underscored the potential of cordycepin as a functional agent in combating fatigue and neurobiological damage resultant from excessive physical exertion (60) (Table 3). Another investigation sought to assess the anti-fatigue and cognitive-enhancing properties of acidic polysaccharides derived from Cordyceps militaris (CMPB) and to elucidate their mechanisms through Nrf2-related signaling pathways. Excessive physical exertion precipitates oxidative stress, inflicts damage upon the central nervous system, and contributes to cognitive decline. Administration of CMPB resulted in a significant enhancement of learning and memory capabilities in murine subjects, alongside a reduction in fatigue-related metabolites and oxidative stress biomarkers. Analytical evaluation of proteins demonstrated an increase in the expression of BDNF, PI3K, Nrf2, and HO-1 within the hippocampus. *In vitro* experimentation revealed that CMPB-b, the bioactive component, augmented antioxidative defense mechanisms by facilitating PI3K/AKT phosphorylation, promoting Nrf2 nuclear translocation, and enhancing HO-1 expression in models of oxidative stress. These results indicated that CMPB mitigates fatigue induced by exercise and cognitive deficits by modulating the Nrf2 pathway, thereby endorsing its prospective utilization in health products aimed at enhancing fatigue resistance (61).

**Table 3:** Mitochondrial energy regulation and anti-fatigue effects of *Cordyceps*.

Model/subjects	Compound/dose	Key findings	Proposed mechanism/pathway	Ref
Mice (forced exercise)	Cordycepin, oral, dose not specified	↑ Endurance, ↑ liver and muscle glycogen, ↓ lactic acid, CK, BUN, oxidative stress; improved cognition	Keap1/Nrf2/HO-1 signaling pathway; increased BDNF	Cheng et al. (60)
Mice (exercise fatigue)	CMPB (acidic polysaccharides), 800 mg/kg	Improved learning & memory, ↓ fatigue metabolites, ↑ BDNF, PI3K, Nrf2, HO-1 expression	PI3K/Akt/Nrf2/HO-1 signaling pathway	Bai et al. (61)
Mice, grip strength test	Ethyl acetate extract (CMEE)	Slight ↑ grip strength, ↑ ATP-related biomarkers, minimal effect on fatigue biomarkers	Enhancement of cellular energy (ATP) production	Choi et al. (62)
Mice, forced swimming	PCM polysaccharide, 40–160 mg/kg/day	↑ swim time, ↓ lactic acid, CK, liver enzymes, MDA; ↑ antioxidant enzymes, glycogen	Antioxidant effects; improved energy metabolism	Xu et al. (63)
Mice, exhaustive swimming	CSP, 100–400 mg/kg	↑ swim time; ↑ SOD, GPx, CAT activities; ↓ MDA, 8-OHdG	Activation of antioxidant enzymes	Yan et al. (64)
Rats, swimming	CS mycelium, 200 mg/kg	↑ endurance 1.8–2.9 fold; ↑ AMPK, PGC-1α, PPAR-δ, GLUT4, VEGF, NRF-2, SOD1, TRX expression	Activation of metabolic regulators and antioxidant response	Kumar et al. (65)
Humans, endurance-trained cyclists	CordyMax Cs-4 tablets, 3 g/day for 5 weeks	No change in VO2peak, ventilatory threshold, or time trial performance	No significant effect on aerobic capacity	Parcell et al. (66)

Choi et al. (62) conducted to elucidate the effects of the ethyl acetate extract of Cordyceps militaris (CMEE) on athletic performance. Although *C. militaris* is recognized for its diverse pharmacological properties, its specific influence on exercise performance remained ambiguous. Mice were administered CMEE, and their grip strength was assessed on a weekly basis. The administration of CMEE resulted in a modest enhancement of grip strength, which was found to be comparable to the effects of red ginseng treatment. Biochemical evaluations revealed that CMEE modulated biomarkers associated with ATP synthesis, while exhibiting negligible effects on markers indicative of muscle fatigue. These findings imply that *C. militaris* may augment exercise performance predominantly through the facilitation of ATP production, rather than through the mitigation of muscle fatigue. This investigation underscored the prospective utility of *C. militaris* as a natural supplement to enhance physical performance via energy metabolic pathways (62). A work sought to assess the anti-fatigue properties of polysaccharides derived from Cordyceps militaris (PCM) employing a forced swimming paradigm in murine subjects. The subjects were administered differing dosages of PCM over a duration of 28 days, subsequently followed by the evaluation of swimming endurance and biochemical markers associated with fatigue. Treatment with PCM resulted in a significant extension of exhaustive swimming duration and a marked reduction in serum concentrations of lactic acid, urea nitrogen, creatine kinase, and indicators of oxidative stress. Furthermore, PCM was found to augment glycogen reserves in both hepatic and muscular tissues while simultaneously enhancing the activities of antioxidant enzymes, which encompassed superoxide dismutase, glutathione peroxidase, and catalase. These findings elucidate that PCM effectively mitigates physical fatigue through the enhancement of energy reserves and the fortification of antioxidant defenses. The results advocated for the potential application of PCM as a functional food or therapeutic modality aimed at improving fatigue resistance (63).

Yan et al. (64) aimed to examine the effects of polysaccharides from *Cordyceps sinensis* (CSP) on oxidative stress induced by exhaustive exercise in mice. Mice were treated with varying doses of CSP for 28 days before undergoing forced swimming. CSP treatment significantly prolonged swimming time and enhanced antioxidant enzyme activities, including glutathione peroxidase (GPx), superoxide dismutase (SOD), and catalase (CAT) in liver, serum, and muscle. Levels of oxidative stress markers like malondialdehyde (MDA) and 8-hydroxy-2′-deoxyguanosine were significantly reduced in treated groups. These findings suggested that CSP effectively mitigates exercise-induced oxidative damage by boosting the body’s antioxidant defenses, supporting its potential as a natural agent to combat physical fatigue and oxidative stress (64). While these preclinical studies demonstrate clear anti-fatigue effects via antioxidant enzyme activation and mitochondrial regulation, they rely entirely on forced-swimming rodent models and indirect blood biomarkers. Translating increased swimming time in a mouse to elite human athletic performance is highly speculative. An increase in mouse swimming endurance does not automatically equate to improved mitochondrial efficiency in humans. Furthermore, jumping from an observed change in a biomarker, such as reduced serum lactic acid or creatine kinase, to a definitive mechanistic conclusion about human performance is a major leap. Direct functional measurements in human trials are required before making concrete ergogenic claims.

A research investigation was conducted to assess the effects and underlying mechanisms of exercise endurance enhancement attributed to the whole mycelium of Cordyceps sinensis (CS) in a rat model. Subjects received supplementation of CS, either in conjunction with or independent of swimming exercise, over a duration of 15 days. The administration of CS resulted in a substantial enhancement of exercise endurance, with an increase in swimming duration reaching up to 2.9-fold in comparison to the placebo group. Molecular investigations revealed that CS markedly upregulated essential metabolic regulators within skeletal muscle (AMPK, PGC-1α, PPAR-δ), alongside genes implicated in angiogenesis, glucose and lactate transport (MCT1, MCT4, GLUT4, VEGF), as well as those associated with antioxidant defense mechanisms (NRF-2, SOD1, TRX). These beneficial effects were observed in both the cohorts subjected to exercise and those that were not, indicating that CS augments endurance by fostering metabolic efficiency and orchestrating antioxidant responses. The results provided substantial support for the potential application of Cordyceps sinensis as a natural exercise mimetic to enhance physical fitness (65).

Another research endeavor sought to evaluate the impact of CordyMax Cs-4 (a Cordyceps sinensis supplementation) on aerobic capacity and endurance performance among trained male cyclists. A cohort of twenty-two cyclists engaged in a five-week supplementation regimen (3 g/day) while preserving their training intensity. Assessments conducted pre- and post-supplementation included measurements of VO₂peak, ventilatory threshold, and a work-based time trial. Findings indicated no statistically significant alterations in VO₂peak or ventilatory threshold within either the supplementation or placebo cohorts. Additionally, performance in the time trial exhibited no notable changes subsequent to supplementation. The study deduced that a five-week regimen of CordyMax Cs-4 supplementation does not enhance aerobic capacity or endurance performance in cyclists who are already endurance-trained (66). Current empirical evidence underscores that bioactive constituents present in Cordyceps, including cordycepin and polysaccharides, enhance mitochondrial bioenergetics and activate antioxidant signaling cascades, particularly the PI3K/NRF2/HO-1 pathway. These physiological effects are associated with a decrease in oxidative stress and a postponement of muscle fatigue onset. Nonetheless, certain investigations indicate minimal or non-existent ergogenic advantages, suggesting that variables such as dosage, duration of supplementation, and individual physiological variability significantly affect the results. Additional mechanistic and clinical investigations are essential to refine supplementation protocols of Cordyceps that are specifically tailored to meet the requirements of athletes.

## 8 Exercise-induced biomarkers: inflammation, oxidative stress, and recovery

**Figure 2:**
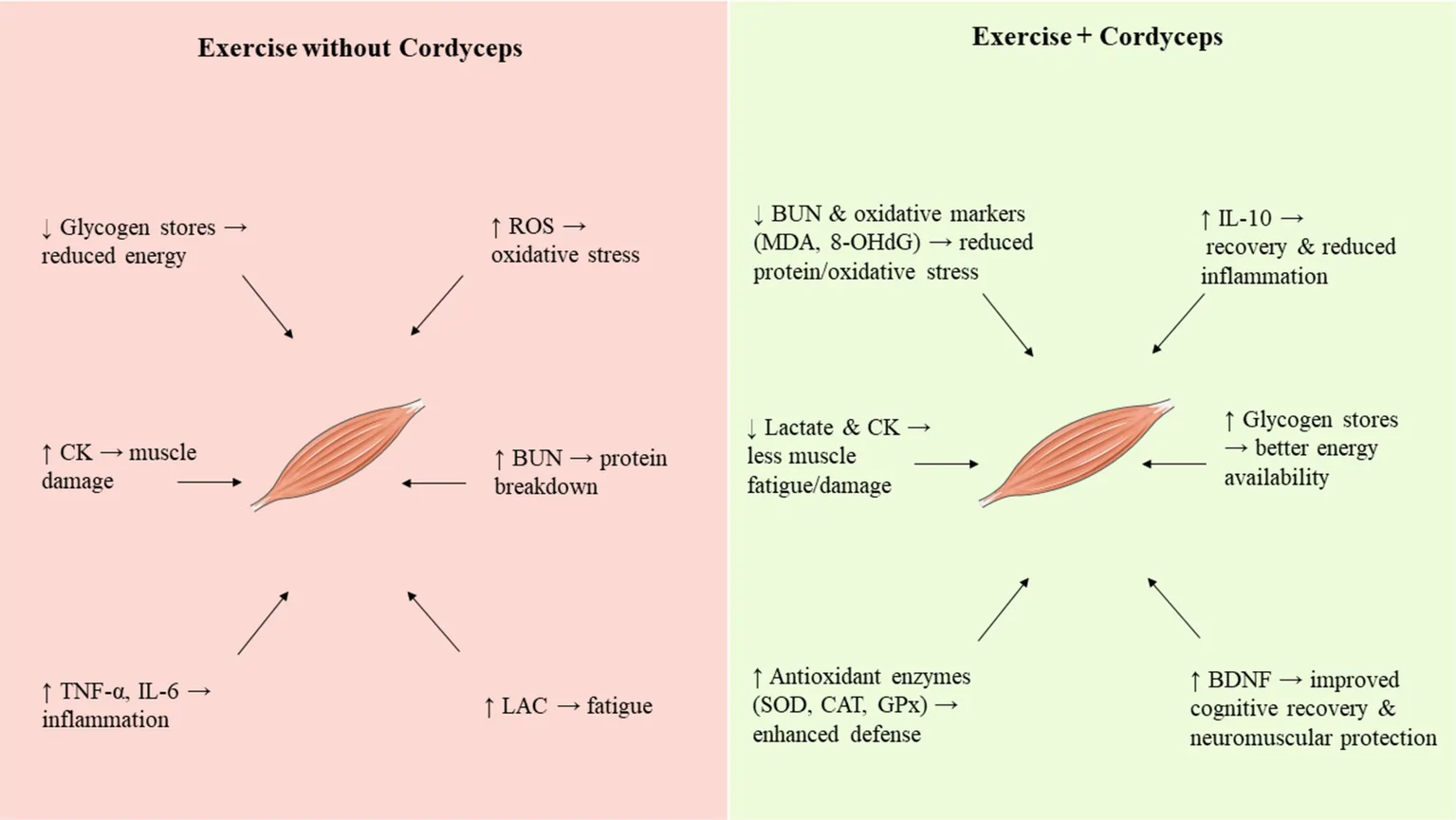
Comparison of exercise responses with and without cordyceps supplementation. Intense exercise elevates lactate, CK, BUN, ROS, and inflammatory cytokines (TNF-α, IL-6), while reducing glycogen. Cordyceps reverses these trends by enhancing SOD, CAT, GPx, BDNF, and IL-10, supporting faster recovery. CK, creatine kinase; BUN, blood urea nitrogen; ROS, reactive oxygen species; TNF-α, tumor necrosis factor-α; IL-6, interleukin-6; SOD, superoxide dismutase; CAT, catalase; GPx, glutathione peroxidase; BDNF, brain-derived neurotrophic factor; IL-10, interleukin-10.

Exercise-induced inflammation and oxidative stress represent pivotal elements that significantly contribute to muscle injury, protracted recovery, and diminished athletic performance. Examining the regulatory effects of Cordyceps-derived supplements on these biomarkers is imperative for elucidating their potential in facilitating recovery and mitigating exercise-related physiological stress. This section systematically reviews empirical evidence regarding the impact of Cordyceps on inflammatory and oxidative pathways, with the objective of guiding nutritional interventions that promote optimal recovery and sustained athletic performance (Figure 2). Nieman et al. (67) conducted to examine the impacts of a mixed beet-derived supplementation (BEET), which comprises nitrates, caffeine, vitamins, and a blend of mushrooms including Cordyceps sinensis, on exercise-induced inflammatory responses in cyclists. Employing a randomized, placebo-controlled, double-blind crossover methodology, 20 cyclists engaged in supplementation for a duration of 2 weeks prior to undertaking rigorous cycling activities. BEET supplementation resulted in a significant elevation of plasma nitrate and nitrite concentrations, as well as an increase in anti-inflammatory oxylipins subsequent to exercise. Proteomic analysis indicated that BEET supplementation diminished the levels of proteins linked to inflammation, encompassing those involved in complement activation and immune system responses. These results implied that a 2-week regimen of BEET supplementation may mitigate exercise-induced inflammatory responses by influencing critical inflammatory proteins and enhancing anti-inflammatory metabolites, thereby endorsing its potential to facilitate recovery in athletes (67) (Table 4).

**Table 4:** The effects of Cordyceps-based supplements on exercise-induced biomarkers: inflammation, oxidative stress, and recovery.

Model/subjects	Compound/dose	Key findings	Proposed mechanism/pathway	Ref
Twenty male and female cyclists	Mixed beet supplement: 212 mg nitrates/day + caffeine, vitamin C, B-vitamins, mushroom blend (Cordyceps + Inonotus)	↑ Plasma nitrate/nitrite, ↑ anti-inflammatory oxylipins (18-HEPE, 4-HDoHE), ↓ inflammation-related proteins post-exercise	Nitrate-related anti-inflammatory effects; complement and immune modulation	Nieman et al. (67)
Mice (*n* = 20/group)	EC and ECC (extruded cereal grains with or without Cordyceps), 5–20 g/kg/day, 30 days oral gavage	↑ Swimming endurance; ↓ blood lactic acid, CK, BUN, MDA; ↑ antioxidant enzymes and glycogen; ECC better than EC	Antioxidant activity; improved energy metabolism via Cordyceps	Zhong et al. (68)
Mice, D-GalN/LPS liver injury model	Cordyceps sinensis, 20–40 mg/kg + exercise (swimming) for 4 weeks	Exercise or CS alone ↓ TNF-α, AST, NO, apoptosis; combined treatment showed no benefit and reduced anti-inflammatory IL-10	Antagonistic interaction between exercise and CS; diminished anti-inflammatory effects combined	Cheng et al. (69)
Mice (ICR)	Six extracts: Cordyceps militaris, Paecilomyces japonica, Phellinus linteus, Ganoderma lucidum, Grifola frondosa, *Panax ginseng* (500 mg/kg/day, 4 weeks)	CM, PJ, GF ↑ swimming time; PJ, GF ↓ plasma lactate and ammonia; no changes in glycogen	Enhanced fat utilization; delayed lactate and ammonia accumulation	Jung et al. (70)
Female soccer players (*n* = 39)	Cordyceps sinensis, 3 g/day + 4-week repeated sprint training under hypoxia or normoxia	↑ Time to exhaustion (TTE); improved repeated sprint ability (mean power, speed) in hypoxia + CS group; no changes in VO2max or oxidative stress markers	Improved peripheral muscle oxygenation; no change in oxidative stress/inflammation markers	Thongsawang et al. (71)

Another investigation sought to assess the anti-fatigue properties of extruded cereal grain products (EC) and the combination of EC with Cordyceps militaris (ECC) in a murine model. Over the span of 30 days, the subjects received varying dosages of EC or ECC and were subsequently evaluated for endurance as well as fatigue-related biochemical markers. Both EC and ECC significantly enhanced swimming endurance and improved biochemical parameters, including lactate dehydrogenase, blood lactic acid, creatine kinase, blood urea nitrogen, and indicators of oxidative stress. ECC, particularly at elevated dosages, exhibited superior anti-fatigue effects in comparison to EC alone, leading to increased glycogen reserves and heightened antioxidant enzyme activities. The results indicated that *C. militaris* in ECC plays a pivotal role in alleviating exercise-induced fatigue, thereby endorsing its potential as a functional food component for the prevention of fatigue (68). A research investigation examined the synergistic effects of physical exercise and supplementation with Cordyceps sinensis (CS) on the development of fulminant hepatic failure induced by D-GalN/LPS in murine models. Experimental groups were administered exercise, pretreatment with CS, or a combination of both prior to the induction of liver injury. Both physical exercise and high-dose CS administered in isolation markedly attenuated liver damage biomarkers, inflammatory cytokines (specifically TNF-α), oxidative stress markers, and hepatocyte apoptosis, while concurrently elevating the levels of the anti-inflammatory cytokine IL-10 and the antioxidant enzyme superoxide dismutase (SOD). Nevertheless, the simultaneous application of exercise and CS did not confer any added protective benefit and instead appeared to mitigate the anti-inflammatory response. These observations indicate that while either exercise or CS alone offers protection against septic hepatic injury, their concurrent use may lead to antagonistic physiological effects. The findings of this study underscore the necessity for caution regarding the concomitant use of Cordyceps sinensis supplements with regular physical exercise, given the potential adverse interactions that could compromise hepatic health (69).

Jung et al. (70) assessed the tonic properties of six medicinal fungi and plant species—Cordyceps militaris (CM), Ganoderma lucidum (GL), Paecilomyces japonica (PJ), Grifola frondosa (GF), Phellinus linteus (PL), and *Panax ginseng* (PG)—on exercise endurance in murine models. The subjects were administered extracts orally for a duration of 4 weeks, and the forced swimming time to the point of exhaustion was quantified alongside various biochemical parameters. The groups receiving CM, PJ, and GF demonstrated significantly prolonged swimming durations in comparison to the control group. The treatments with PJ and GF resulted in reductions in plasma triglyceride, lactate, and ammonia concentrations, suggesting enhanced lipid utilization and a postponement of fatigue onset. No substantial disparities were detected in plasma glucose or glycogen levels across the groups, with the exception of PG’s influence on glucose. The findings indicated that the extracts of PJ and GF augment endurance capacity by facilitating energy metabolism while concurrently diminishing fatigue-related metabolites (70). A research investigation assessed the impact of Cordyceps sinensis (CS) supplementation on various hematological parameters, indicators of oxidative stress, nitric oxide synthesis, aerobic performance, and repeated sprint ability (RSA) among female soccer athletes undergoing training in hypoxic (RSH) or normoxic (RSN) conditions. A total of 39 athletes were allocated into either CS or placebo cohorts and underwent a regimen of repeated sprint training for a duration of 4 weeks. The administration of CS supplementation resulted in a significant enhancement in time to exhaustion (TTE) irrespective of the training environment, yet it did not influence VO2max or hematological outcomes. Significantly, mean power output and velocity during sprint assessments exhibited notable improvement solely in the hypoxia plus CS cohort (RSH-C), which was associated with an increase in muscle deoxygenation while maintaining stable levels of oxidative stress and inflammatory markers. The results indicated that CS supplementation during hypoxic sprint training may facilitate improvements in RSA by optimizing peripheral muscle oxygen utilization, without effecting changes in aerobic capacity or oxidative stress (71). Overall, available empirical evidence indicates that the supplementation of Cordyceps may mitigate exercise-induced inflammation and oxidative stress through the enhancement of antioxidant mechanisms and the modulation of immune responses. Nevertheless, the magnitude of these advantages is contingent upon various factors, including the modality of exercise, the formulation of the supplement, and individual variability among subjects. Certain investigations also emphasize the potential for detrimental effects under particular pathological contexts, thereby highlighting the necessity for tailored therapeutic strategies. Rigorous and meticulously controlled trials are imperative to elucidate optimal dosage regimens and the long-term effects on biomarkers associated with recovery.

## 9 Effects on body composition and physiological adaptation to exercise

Body composition, encompassing the equilibrium between lean muscle tissue and adipose tissue, is instrumental in determining athletic efficacy and holistic health. Grasping the mechanisms by which Cordyceps-derived supplements affect physiological changes in response to exercise and influence body composition is crucial for formulating efficacious nutritional approaches within the realm of precision sports nutrition. This section scrutinizes the empirical evidence regarding the impacts of Cordyceps on the enhancement of muscle hypertrophy, the attenuation of fat mass, and the advancement of training adaptations across varied athletic demographics. A research investigation was conducted to assess the impact of Cordyceps sinensis (CS) supplementation on various hematological parameters, oxidative stress indicators, nitric oxide synthesis, aerobic performance, and repeated sprint ability (RSA) among female soccer athletes engaged in training under hypoxic (RSH) or normoxic (RSN) conditions. A total of 39 athletes were systematically allocated to either the CS supplementation or placebo cohort and underwent a regimen of 4 weeks of repeated sprint training. The administration of CS supplementation resulted in a significant enhancement in time to exhaustion (TTE), irrespective of the training environment, yet it did not yield alterations in VO2max or hematological metrics. Of particular significance, mean power output and velocity during sprint assessments exhibited substantial improvements exclusively within the hypoxia plus CS group (RSH-C), which was associated with augmented muscle deoxygenation, while no modifications were observed in oxidative stress or inflammatory markers. The results indicated that CS supplementation during hypoxic sprint training could potentially augment RSA through improved peripheral muscle oxygen utilization, without inducing changes in aerobic capacity or oxidative stress (72) (Table 5). Liao et al. (73) assessed the implications of integrating Rhodiola crenulata and Cordyceps sinensis (RC) supplementation with an 8-week endurance training regimen in a cohort of young, sedentary adults. A total of 14 subjects were assigned randomly to one of two experimental conditions: endurance training supplemented with RC (20 mg/kg/day) or a placebo control. Both experimental groups demonstrated enhancements in endurance capacity as well as glycemic regulation. Nonetheless, the RC supplementation group manifested markedly superior advancements in body composition, which included a decrease in body weight, body mass index (BMI), and fat mass, in addition to an augmentation in muscle mass when contrasted with the placebo group. No statistically significant disparities were observed regarding blood lipid profiles or systemic markers of oxidative stress between the experimental cohorts. These findings exhibited that RC supplementation might marginally augment the beneficial effects of endurance training on body composition, albeit it does not appear to influence lipid metabolism or oxidative stress levels in young sedentary individuals (73).

**Table 5:** The effects of Cordyceps-based supplements on body composition and physiological adaptation to exercise.

Model/subjects	Compound/dose	Key findings	Proposed mechanism/pathway	Ref
Twenty-one active college-aged men; 14 weeks concurrent training (resistance + HIIT)	Multi-ingredient supplement containing *Rhodiola rosea* and Cordyceps sinensis; dose not specified	↑ weekly sprint performance, ↑ bench press volume, ↑ total workload; ↔ overall body composition, strength gains, hormone profiles (no difference vs. placebo)	Possible acute improvements in energy metabolism and fatigue resistance; no sustained systemic hormonal changes	Kreipke et al. (72)
Fourteen young sedentary adults (8 M/6F); 8 weeks endurance training	Rhodiola crenulata + Cordyceps sinensis; 20 mg/kg/day	↑ body weight, ↑ BMI, ↑ muscle mass, ↓ fat mass; ↔ oxidative stress and metabolic biomarkers	Anti-inflammatory and antioxidant effects; modulation of body fat and muscle mass likely through mitochondrial energy regulation	Liao et al. (73)
Twenty-eight healthy adults (age 22.7 ± 4.1); acute (1 week) and chronic (3 weeks) supplementation	Cordyceps militaris mushroom blend; 4 g/day	After 3 weeks: ↑ VO2max (+4.8 ml·kg^−1^·min^−1^), ↑ time to exhaustion (+69.8 s), ↑ ventilatory threshold; after 1 week: ↔ no significant changes	Enhancement of mitochondrial function and oxygen utilization; improved aerobic capacity and fatigue resistance via energy metabolism pathways	Hirsch et al. (74)
Eighteen male subjects; 2 weeks altitude training at 2200 m	Rhodiola crenulata (1,400 mg) + Cordyceps sinensis (600 mg) daily	↑ exhaustive run time (+5.7%), ↓ parasympathetic activity decline (better preservation), ↔ hematological adaptation, ↓ erythropoietin levels vs. placebo	Maintenance of autonomic nervous system balance; enhanced physiological adaptation to hypoxia; improved mitochondrial efficiency and reduced fatigue	Chen et al. (75)

An investigation was conducted to examine the implications of a mushroom blend containing Cordyceps militaris on performance during high-intensity exercise across 1 and 3-week periods. A total of 28 young adults were enrolled in a randomized, double-blind, placebo-controlled study. Participants ingested 4 g/day of either the mushroom blend or a placebo and underwent exercise assessments that measured VO2max, time to exhaustion (TTE), ventilatory threshold (VT), and power output. Following the 1-week period, no statistically significant differences were noted between the groups. Nevertheless, after 3 weeks, the group receiving the mushroom blend exhibited a notable enhancement in VO2max (+4.8 mL·kg^−1^·min^−1^), an improvement in TTE (+69.8 s), and an increase in VT (+0.7 L·min^−1^), whereas the placebo group demonstrated no alterations. These results imply that prolonged supplementation with Cordyceps militaris may contribute to improved tolerance during high-intensity exercise and augmented aerobic capacity (74).

Chen et al. (75) assessed the impact of Rhodiola crenulata and Cordyceps sinensis (RC) supplementation on the enhancement of aerobic performance resulting from high-altitude training. Eighteen male subjects engaged in a two-week regimen of endurance training at an altitude of 2,200 m while receiving either RC (1,400 mg R + 600 mg C daily) or a placebo intervention. In comparison to the placebo cohort, the RC group demonstrated a statistically significant enhancement in exhaustive running duration (+5.7% vs. +2.2%) and a superior maintenance of parasympathetic nervous system functionality (−41% vs. −51%). Both groups exhibited similar increments in red blood cell count, hematocrit levels, and hemoglobin concentration, although erythropoietin (EPO) levels were notably elevated in the placebo cohort. These findings indicate that RC supplementation during altitude training may augment aerobic capacity, potentially through the facilitation of autonomic balance and the acceleration of physiological adaptations (75). The studies that have been reviewed suggest that supplementation with Cordyceps, particularly in conjunction with regimented training regimes, can significantly influence body composition by facilitating the increase of lean muscle mass and the reduction of adipose tissue. Furthermore, Cordyceps seems to enhance physiological adaptations to strenuous environments, including those encountered during high-altitude training. Nonetheless, the variability observed in study methodologies, supplementation regimens, and participant demographics indicates that individualized dosing and timing are of paramount importance. Subsequent research endeavors should prioritize long-term interventions and mechanistic explorations to comprehensively clarify the role of Cordyceps in the modulation of body composition.

## 10 Athletic performance outcomes across exercise modalities

**Figure 3:**
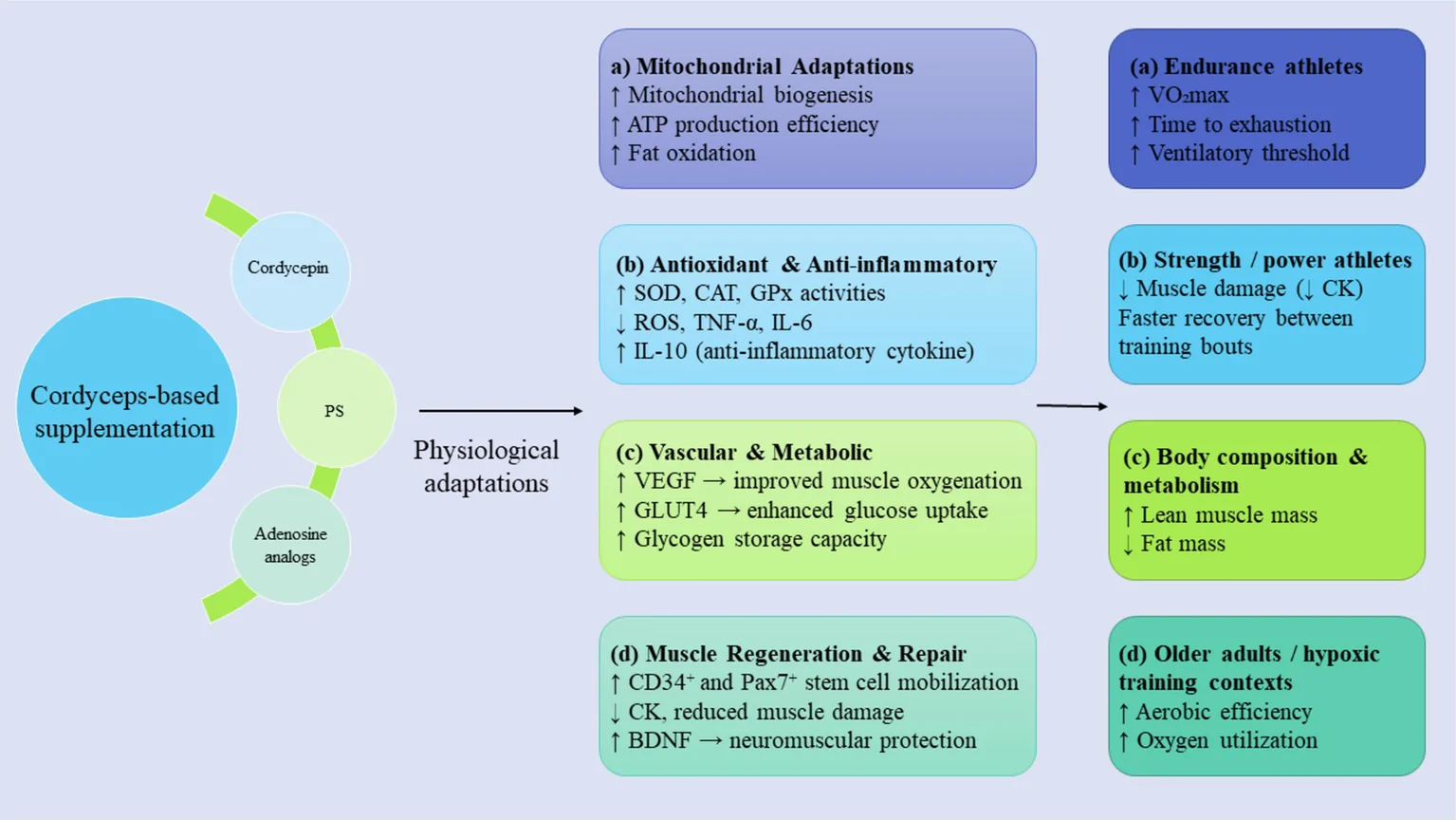
Integrated effects of *Cordyceps* supplementation on physiology and performance. Enhanced AMPK/PGC-1α activity, antioxidant capacity, VEGF-mediated oxygen delivery, and stem-cell recruitment lead to improved VO₂max, endurance, recovery, lean mass, and reduced muscle damage (↓ CK). AMPK, AMP-activated protein kinase; PGC-1α, peroxisome proliferator-activated receptor γ coactivator-1α; PS, polysaccharide; VEGF, vascular endothelial growth factor; CK, creatine kinase; VO₂max, maximal oxygen uptake.

The performance of athletes is shaped by a multifaceted interaction of physiological, biochemical, and environmental determinants. An examination of the effectiveness of Cordyceps-derived supplements across diverse exercise modalities yields critical insights into their capacity to augment endurance, strength, and recovery in athletic populations. This section consolidates contemporary evidence regarding the impact of Cordyceps on performance metrics across varying demographics, exercise intensities, and environmental contexts, thereby informing tailored supplementation approaches within the realm of sports nutrition (Figure 3). An investigation assessed the impact of Cordyceps militaris (CM) mycelium extract on anemia and muscle damage biomarkers in long-distance runners throughout a 16-week pre-season training period. The study involved participants (n = 22) who were administered either the CM extract or a placebo. The group receiving CM demonstrated a statistically significant elevation in serum ferritin levels at both weeks 4 and 8, alongside notable enhancements in hemoglobin and hematocrit concentrations by week 8, thereby indicating improved iron status maintenance. Furthermore, the levels of creatine kinase—initially heightened in both cohorts—exhibited a statistically significant decrease in the CM group by week 16, signifying a reduction in muscle damage. These results shown that CM extract may serve as a preventative measure against anemia and provide protection against muscle injury during rigorous training regimens in endurance athletes (76) (Table 6).

**Table 6:** The effects of Cordyceps-based supplements on athletic performance outcomes across exercise modalities.

Model/subjects	Compound/dose	Key findings	Proposed mechanism/pathway	Ref
Twenty-two long-distance runners (11 CM group, 11 placebo), 16 weeks pre-season training	Cordyceps militaris mycelium extract, dose not specified	↑ serum ferritin (4 and 8 weeks), ↑ hemoglobin & hematocrit (8 weeks), ↓ creatine kinase (16 weeks) vs. placebo	Suppression of iron storage decline (serum ferritin), protection against muscle damage, reduced muscle injury	Nakamura et al. (76)
Fourteen young adults (24 ± 0.8 y), double-blind crossover	Cordyceps sinensis, 1 g before exercise	↓ necrotic cell infiltration (3 h post-exercise), ↑ CD34 + cells (3 h & 24 h), ↑ Pax7 + cells (4-fold)	Accelerated stem cell recruitment and muscle regeneration; immune modulation and faster resolution of muscle damage	Dewi et al. (77)
Ninety-six healthy physical education students (26.3 ± 3.2 y), 28–33 days	Commercial supplement of Cordyceps sinensis + Ganoderma lucidum, doses not specified	↔ VO2max, heart rate, anaerobic power, fatigue index; borderline ↓ resting HR (not significant)	No significant physiological effect on aerobic or anaerobic capacity in healthy young adults	Tsuk et al. (78)
Twenty healthy elderly (50–75 y), 12 weeks	Cs-4 (Cordyceps sinensis), 333 mg, 3x daily	↑ metabolic threshold (+10.5%), ↑ ventilatory threshold (+8.5%), ↔ VO2max	Improved aerobic efficiency and exercise capacity via enhanced metabolic and ventilatory thresholds	Chen et al. (79)
Twenty-four recreational athletes (21.1 ± 1.9 y), 3 weeks	Proprietary blend including Cordyceps sinensis (18 g total), 30 min pre-exercise	↑ VO2max (+10.3%), ↑ critical velocity (+2.9%), ↑ lean body mass; ↓ % body fat (not significant)	Enhanced aerobic capacity, training volume, and body composition; improved energy metabolism	Smith et al. (80)
Eight male cyclists, double-blind	Cordyceps sinensis + *Rhodiola rosea*, doses not specified	↔ muscle oxygen saturation (Sto2), VO2max, ventilatory threshold, time to exhaustion	No significant effect on muscle oxygenation or exercise performance variables	Colson et al. (81)
Seventeen amateur cyclists, 2 weeks	Cordyceps sinensis (1,000 mg) + *Rhodiola rosea* (300 mg) + other ingredients	↔ VO2max, time to exhaustion, peak power output, ventilatory threshold	Insufficient duration/dose to improve cycling performance	Earnest et al. (82)
Thirty-six male sedentary subjects, 2 weeks	Cordyceps sinensis powder, dose not specified	↓ VO2/kg, VE, lactic acid during recovery; altered catecholamines and cortisol	Enhanced energy generation and anti-fatigue ability through improved energy metabolism	Nagata et al. (83)
Thirty amateur marathoners, randomized double-blind placebo-controlled, 12 weeks	Cordyceps sinensis, 2 g/day	↓ heart rate at same submaximal intensity (8 weeks), ↑ aerobic performance (12 weeks)	Improved cardiovascular efficiency leading to enhanced aerobic capacity	Savioli et al. (84)

Dewi et al. (77) examined the impact of Cordyceps sinensis supplementation on the process of muscle regeneration subsequent to high-intensity interval exercise (HIIE) involving 14 young adult subjects. Participants were administered either 1 g of Cordyceps or a placebo prior to engaging in HIIE, with muscle biopsies collected both pre- and post-exercise. The supplementation with Cordyceps was found to significantly diminish necrotic cell infiltration at the three-hour mark following exercise and to expedite the mobilization of CD34 + stem cells (+51%) into muscle tissue. This early mobilization was associated with a four-fold augmentation in Pax7 + muscle stem cells that play a crucial role in regeneration. Furthermore, VEGF mRNA expression levels exhibited a two-fold increase 3 h after HIIE, indicating an enhanced vascular response. These findings represent the inaugural human evidence suggesting that Cordyceps sinensis facilitates muscle repair following strenuous exercise by fostering early stem cell activation and mitigating tissue damage (77). Furthermore, the review elucidates the impacts on body composition, encompassing the preservation of lean mass, the enhancement of fat metabolism, and the remodeling of skeletal muscle, in conjunction with advancements in maximal oxygen uptake and anaerobic threshold. Finally, it delineates the existing knowledge gaps, methodological constraints, and prospective research trajectories to facilitate the translation of findings from experimental and clinical investigations into evidence-based practices within athletic training regimens. This review posits Cordyceps as a promising natural ergogenic aid that offers a plethora of benefits pertaining to energy metabolism, fatigue resistance, body composition, and performance outcomes (78).

Another work examined the impact of Cordyceps sinensis (Cs-4) supplementation on exercise performance among 20 healthy elderly individuals (ages 50–75). Participants received 333 mg of Cs-4 or a placebo three times per day over a duration of 12 weeks. The evaluation of exercise performance was conducted through maximal incremental cycling assessments accompanied by breath-by-breath analysis. Upon completion of the 12-week period, the Cs-4 cohort exhibited statistically significant enhancements in metabolic threshold (+10.5%) and ventilatory threshold (+8.5%), thereby indicating improved submaximal exercise capacity. Conversely, no statistically significant alterations were detected within the placebo cohort, and VO₂max remained constant across both groups. These results implied that Cs-4 supplementation may augment aerobic efficiency and exercise tolerance in older adults, notwithstanding the absence of an increase in maximal oxygen uptake (79). Smith et al. (80) assessed the impacts of a pre-exercise nutritional supplement comprising Cordyceps sinensis and additional constituents (GT) in conjunction with a regimen of 3 weeks of high-intensity interval training (HIIT) involving 24 recreational athletes. The participants ingested either GT or a placebo prior to each training and assessment session. Both cohorts exhibited enhancements in VO₂max; however, the GT cohort demonstrated a more pronounced augmentation (+10.3% versus +2.9%) alongside a statistically significant elevation in critical velocity. The training volume was 11.6% greater in the GT cohort, and a significant increase in lean body mass was observed exclusively in the GT group. Although both groups experienced decreases in body fat, these changes were not statistically significant. Improvements in anaerobic running capacity were noted in both cohorts, with a more substantial enhancement occurring in the placebo group. Collectively, the integration of GT supplementation with HIIT resulted in a more pronounced enhancement of aerobic fitness, training volume, and lean body mass compared to HIIT conducted in isolation (80).

Another investigation examined the impact of a combined Cordyceps sinensis and *Rhodiola rosea* (Cs-Rr) supplementation on muscle oxygen saturation (StO₂) and exercise performance among eight male subjects. Participants were administered either the Cs-Rr formulation or a placebo and underwent two maximal exercise tests to the point of exhaustion on a cycle ergometer. Principal performance indicators—including StO₂ slope, StO₂ threshold, VO₂max, ventilatory threshold, and duration until exhaustion—revealed no statistically significant variations between or within the groups. The results indicated that short-term Cs-Rr supplementation does not exert a meaningful influence on circulatory oxygen dynamics or aerobic performance during maximal exercise in healthy males (81). A study was conducted to assess the impact of a commercially available herbal supplement—comprising Cordyceps sinensis (CS-4), *Rhodiola rosea*, along with additional compounds—on cycling performance and oxygen uptake in a cohort of 17 amateur cyclists. Participants underwent graded cycling assessments both prior to and subsequent to a 14-day supplementation protocol (4-day loading phase followed by 11-day maintenance phase). No statistically significant alterations were detected either between or within the groups with respect to peak VO₂, time to exhaustion, peak power output, heart rate, lactate thresholds, ventilatory threshold, or exercise efficiency. These findings suggest that a 2-week supplementation regimen utilizing this formulation does not yield enhancements in aerobic performance or physiological indicators of endurance among trained cyclists (82).

Nagata et al. (83) investigation examined the ramifications of cultured Cordyceps sinensis (Cs) supplementation on physiological responses to exhaustive running among 36 sedentary male participants over a duration of 2 weeks. The subjects were administered either Cs or a placebo, and an array of bio-signals was assessed, encompassing VO₂, ventilation (VE), heart rate, blood pressure, lactate, urinary catecholamines (CA: adrenaline, noradrenaline, dopamine), and stress hormones (17-OHCS, 17-KS-G). In comparison to the placebo group, the Cs cohort exhibited significantly diminished VO₂/kg, VE, and lactate concentrations during the recovery phase, thereby signifying enhanced exercise efficiency. Additionally, hormonal indicators displayed significant variations, with Cs supplementation linked to modified catecholamine and cortisol-related reactions. These results implied that short-term supplementation with Cordyceps sinensis may potentiate energy metabolism and mitigate fatigue during extended exercise in untrained individuals (83). A research investigation evaluated the impact of Cordyceps sinensis supplementation on aerobic performance among 30 recreational marathon runners. The subjects ingested 2 g/day of Cordyceps for a duration of 12 weeks. Findings indicated a decrease in heart rate at the same submaximal exercise intensity following 8 weeks, along with a notable enhancement in aerobic performance by the conclusion of week 12. These results indicated that Cordyceps sinensis may improve cardiovascular efficiency and aerobic capacity in physically active individuals during an extended supplementation period (84). Overall, the empirical evidence indicates that supplementation with Cordyceps has the potential to enhance endurance capacity, aerobic metabolic function, and recovery times across various groups of athletes, including long-distance runners, cyclists, and older populations. Moreover, the presence of synergistic effects when combined with other ergogenic aids, such as caffeine and creatine, has been documented. However, the discrepancies observed in research outcomes underscore the necessity for personalized strategies that take into account variables such as training status, formulation of supplements, and environmental stressors. Additional large-scale, methodologically rigorous clinical trials are essential to delineate definitive protocols aimed at optimizing athletic performance through Cordyceps supplementation.

### 10.1 Limitations in current research and future directions

1. Primary drivers vs. downstream physiological consequences

A recurring challenge in evaluating ergogenic aids is the tendency to conflate primary molecular drivers with downstream physiological consequences. For example, a reduction in systemic fatigue or improved recovery markers is often a secondary downstream consequence of cellular osmoprotection and enhanced hydration (in the case of betaine), or preserved central catecholamines during acute stress (in the case of tyrosine). These systemic improvements should not be misattributed to direct, primary modifications of mitochondrial respiratory chain kinetics or metabolic rate unless direct functional assays (e.g., high-resolution respirometry) have confirmed such direct interactions.

1. Methodological heterogeneity and translational gaps

It is important to acknowledge several critical limitations within the current body of literature. A significant portion of the existing evidence is derived from studies utilizing small human sample sizes or preclinical animal models (such as forced-swimming rodents), which do not seamlessly translate to elite human athletic performance. Furthermore, profound methodological heterogeneities exist across different studies—encompassing variations in raw species of *Cordyceps* (*C. sinensis* vs. *C. militaris*), cultivation environments, extraction methodologies, intervention durations, participant fitness levels, and specific exercise protocols. Consequently, while current findings suggest that these interventions may contribute to favorable metabolic shifts, their definitive efficacy and exact biochemical mechanisms remain to be fully established *in vivo*.

1. Specific gaps in the proposed triadic model

Mechanistically, the mitochondrial–methylation–neurotransmitter interactions proposed in this review remain largely conjectural in human subjects. In particular, the interactive effects concerning ATP production, methylation-dependent synthesis of creatine, and central catecholamine-mediated modulation of fatigue have not yet been characterized within controlled co-supplementation trials. Furthermore, the pharmacokinetic and pharmacodynamic profiling for these combined strategies is virtually nonexistent, thereby limiting our ability to optimize dosages to achieve true synergistic effects. Finally, a profound cohort bias severely limits the clinical translation of the current literature. The overwhelming majority of human trials have exclusively evaluated young, healthy, physically active male subjects. Consequently, the applicability of this triadic model to older adults or individuals with metabolic disorders remains entirely speculative and grounded purely in theoretical biological plausibility. Geriatric and metabolic-compromised clinical populations exhibit marked differences in baseline mitochondrial respiratory capacity, altered one-carbon enzyme kinetics, and degraded baseline neurotransmitter synthesis. Future research must prioritize dedicated clinical trials in these specific understudied populations rather than relying on unverified extrapolations from elite youth athletic data. To resolve these gaps, subsequent investigations must move away from relying on indirect blood biomarkers and instead emphasize meticulously designed human trials. These future directions should integrate:

- Standardized formulations with clearly characterized active compound concentrations.
- Heterogeneous cohorts including female athletes and clinical populations.
- Multi-omic methodologies (genomics, metabolomics, and microbiome evaluations) to clarify individual responder profiles and identify predictive biomarkers.
- Longitudinal evaluations of direct, functional human performance and recovery metrics.

## 11 Conclusion

The integration of Cordyceps, betaine, and tyrosine represents a compelling biological possibility for targeting mitochondrial bioenergetics, methylation pathways, and central neurotransmitters. However, it must be stated clearly that this synergistic model is currently an integrative mechanistic hypothesis rather than a proven scientific fact. While the proposed model underscores a theoretical framework for enhancing ATP synthesis, bolstering muscle power, and mitigating fatigue, the current body of evidence is severely constrained by formulation heterogeneity, a lack of human co-supplementation trials, and a reliance on indirect biomarkers rather than direct functional measurements of human performance. By transitioning from speculative, narrative claims to rigorous, translational research that utilizes standardized protocols and multi-omic analyses, future research can substantiate whether this triadic framework can safely serve as a foundation for precision performance nutrition and metabolic health interventions.
